# A Genome‐Wide Association Study Meta‐Analysis of Alpha Angle Suggests Cam‐Type Morphology May Be a Specific Feature of Hip Osteoarthritis in Older Adults

**DOI:** 10.1002/art.42451

**Published:** 2023-04-09

**Authors:** Benjamin G. Faber, Monika Frysz, April E. Hartley, Raja Ebsim, Cindy G. Boer, Fiona R. Saunders, Jennifer S. Gregory, Richard M. Aspden, Nicholas C. Harvey, Lorraine Southam, William Giles, Christine L. Le Maitre, J. Mark Wilkinson, Joyce B. J. van Meurs, Eleftheria Zeggini, Timothy Cootes, Claudia Lindner, John P. Kemp, George Davey Smith, Jonathan H. Tobias

**Affiliations:** ^1^ Musculoskeletal Research Unit, Translational Health Sciences, and Medical Research Council Integrative Epidemiology Unit, Population Health Sciences, Bristol Medical School University of Bristol UK; ^2^ Medical Research Council Integrative Epidemiology Unit, Population Health Sciences, Bristol Medical School University of Bristol UK; ^3^ Division of Informatics, Imaging and Data Science The University of Manchester UK; ^4^ Department of Internal Medicine, Erasmus MC University Medical Center Rotterdam The Netherlands; ^5^ Centre for Arthritis and Musculoskeletal Health University of Aberdeen UK; ^6^ Medical Research Council Lifecourse Epidemiology Centre, University of Southampton, UK, and NIHR Southampton Biomedical Research Centre University of Southampton and University Hospital Southampton NHS Foundation Trust UK; ^7^ Institute of Translational Genomics, Helmholtz Zentrum München–German Research Center for Environmental Health Neuherberg Germany; ^8^ Department of Oncology and Metabolism The University of Sheffield UK; ^9^ Biomolecular Sciences Research Centre Sheffield Hallam University UK; ^10^ Department of Internal Medicine and Department of Orthopaedics & Sports Medicine, Erasmus MC Rotterdam The Netherlands; ^11^ Institute of Translational Genomics, Helmholtz Zentrum München–German Research Center for Environmental Health, Neuherberg, Germany, and TUM School of Medicine Technical University of Munich and Klinikum Rechts der Isar Germany; ^12^ Medical Research Council Integrative Epidemiology Unit, Population Health Sciences, Bristol Medical School, University of Bristol, UK, and The University of Queensland Diamantina Institute and Institute for Molecular Bioscience, The University of Queensland Queensland Australia

## Abstract

**Objective:**

To examine the genetic architecture of cam morphology using alpha angle (AA) as a proxy measure and conduct an AA genome‐wide association study (GWAS) followed by Mendelian randomization (MR) to evaluate its causal relationship with hip osteoarthritis (OA).

**Methods:**

Observational analyses examined associations between AA measurements derived from hip dual x‐ray absorptiometry (DXA) scans from the UK Biobank study and radiographic hip OA outcomes and subsequent total hip replacement. Following these analyses, an AA GWAS meta‐analysis was performed (N = 44,214) using AA measurements previously derived in the Rotterdam Study. Linkage disequilibrium score regression assessed the genetic correlation between AA and hip OA. Genetic associations considered significant (*P* < 5 × 10^−8^) were used as AA genetic instrument for 2‐sample MR analysis.

**Results:**

DXA‐derived AA showed expected associations between AA and radiographic hip OA (adjusted odds ratio [OR] 1.63 [95% confidence interval (95% CI) 1.58, 1.67]) and between AA and total hip replacement (adjusted hazard ratio 1.45 [95% CI 1.33, 1.59]) in the UK Biobank study cohort. The heritability of AA was 10%, and AA had a moderate genetic correlation with hip OA (r_g_ = 0.26 [95% CI 0.10, 0.43]). Eight independent genetic signals were associated with AA. Two‐sample MR provided weak evidence of causal effects of AA on hip OA risk (inverse variance weighted OR 1.84 [95% CI 1.14, 2.96], *P* = 0.01). In contrast, genetic predisposition for hip OA had stronger evidence of a causal effect on increased AA (inverse variance weighted β = 0.09 [95% CI 0.04, 0.13], *P* = 4.58 × 10^−5^).

**Conclusion:**

Expected observational associations between AA and related clinical outcomes provided face validity for the DXA‐derived AA measurements. Evidence of bidirectional associations between AA and hip OA, particularly for risk of hip OA on AA, suggests that hip shape modeling secondary to a genetic predisposition to hip OA contributes to the well‐established relationship between hip OA and cam morphology in older adults.

## INTRODUCTION

Cam morphology describes a nonspherical femoral head and has been associated with hip osteoarthritis (OA) (1, 2). Longitudinal studies have shown that cam morphology precedes hip OA, and from this finding, causation has been inferred (2, 3), prompting research into the benefits of surgical correction (4, 5). That said, observational studies showing temporal associations are still limited by confounding, hindering causal inferences (1, 6, 7).

The alpha angle (AA), a measure of femoral head sphericity, is widely used to define cam morphology when exceeding a prespecified limit, which varies between studies (4, 8). Cam morphology may develop in adolescence due to anterolateral femoral head offset or increased impact as the growth plate fuses, leading to greater bone deposition (9, 10). However, a similar morphology may also develop in later life as a consequence of modeling changes occurring as part of the osteoarthritic process (11). The Croft scoring system for radiographic hip OA specifically recognizes abnormal hip shape as the most advanced stage of hip OA (12).

Femoroacetabular impingement (FAI) has been proposed to explain the causal pathway between cam morphology and hip OA (13). FAI syndrome encompasses hip pain coexistent with cam morphology on imaging and specific examination findings (14). FAI syndrome is seen predominantly in younger adults before the onset of hip OA. Clinical trials of surgical interventions to remove cam lesions in this population have demonstrated an improvement in short‐term activities of daily living and hip‐related quality of life (4, 5). As well as potentially improving hip pain, these surgical procedures have been suggested to prevent the development or progression of hip OA (5).

One method to derive causal inferences from observational data is Mendelian randomization (MR), which uses genetic loci as instrumental variables and largely removes the effects of confounding and reverse causation (15). The UK Biobank study, a cohort study of adults 40–69 years of age at its inception, provides a large sample size required to study the relationship between hip shape and hip OA using MR (16, 17). In this study, we aimed to provide face validity for our novel automated method for deriving AAs from hip dual x‐ray absorptiometry (DXA) scans in the UK Biobank study by confirming expected relationships with hip OA, to perform an AA genome‐wide association study (GWAS) meta‐analysis to establish the genetic architecture of cam morphology, and to use MR analysis based on identified genetic instruments to establish whether there is a causal relationship between increased AA and hip OA.

## PATIENTS AND METHODS

### Alpha angle determination

**Figure 1:**
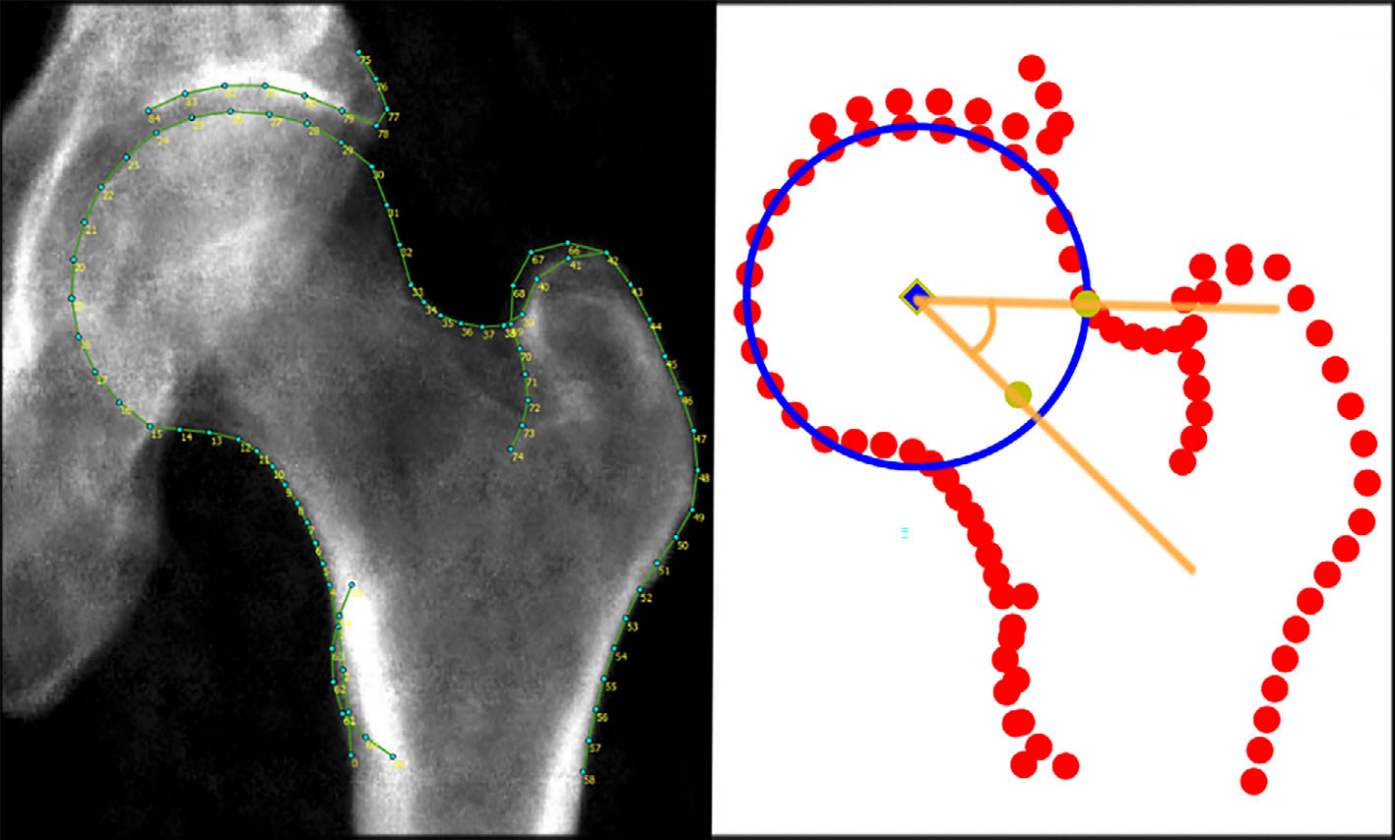
Automatic calculation of the alpha angle in the UK Biobank cohort. Left, Representative UK Biobank dual x‐ray absorptiometry image with outline points marked and lines connecting the points. Right, The same points visualized in Python, where a circle of best fit was plotted, and the alpha angle (indicated by yellow lines) was calculated from the femoral neck midpoint and the point at which the femoral neck intersects the circle. In this individual, the alpha angle was determined to be 41.7°.

This study included UK Biobank participants 44–82 years of age with a left hip DXA scan (Lunar iDXA; GE Healthcare). Outline points were automatically placed around each hip, and features of radiographic hip OA were measured semi‐automatically as previously described (16, 18, 19). AA was estimated using the outline points that excluded identified osteophytes (Figure 1) and a previously published Python code (20, 21). This study also included individuals ≥45 years of age from the Rotterdam Study who had an AA measured from anteroposterior pelvic radiographs using similar methods (22) (see the Supplementary Methods, available on the *Arthritis & Rheumatology* website at https://onlinelibrary.wiley.com/doi/10.1002/art.42451).

Ethics approval was given by the appropriate body for each study. The National Information Governance Board for Health and Social Care and Northwest Multi‐Centre Research Ethics Committee (approval no. 11/NW/0382) and the UK Biobank Ethics Advisory committee gave approval for all work in this study undertaken using UK Biobank data (UK Biobank application no. 17295). The South Yorkshire and North Derbyshire Musculoskeletal Biobank ethics committee gave approval (REC reference no. 20/SC/0144) for the immunohistochemistry experiments in this study. The Medical Ethics Committee of the Erasmus MC (registration no. 02.1015) and the Dutch Ministry of Health, Welfare and Sport (Population Screening Act WBO, license no. 1071272‐159521‐PG) gave approval for all work in this study undertaken using data from the Rotterdam Study. The Rotterdam Study has been entered into the Netherlands National Trial Register and into the World Health Organization International Clinical Trials Registry Platform under shared catalog number NTR6831. All participants provided informed consent for this study.

### Outcome measures of OA and observational associations in the UK Biobank cohort

UK Biobank participants were asked whether they had hip pain for >3 months via questionnaire on the same day as their DXA scan. Hospital‐diagnosed hip OA was based on hospital episode statistics data, as was total hip replacement (18). Logistic regression was used to examine associations between AA and clinical outcomes, except for association between AA and total hip replacement, which was examined using Cox proportional hazards models. Further sensitivity analyses were performed, which defined cam morphology as an AA ≥60° (see Supplementary Methods, available at https://onlinelibrary.wiley.com/doi/10.1002/art.42451).

### Alpha angle GWAS

AA measurements were standardized to create a Z score (SD = 1; mean = 0). Subsequently, a GWAS meta‐analysis of AAs was conducted between a GWAS performed in each study (38,173 individuals from the UK Biobank study, 2,970 individuals from Rotterdam Study I, 1,817 individuals from Rotterdam Study II, and 1,254 individuals from Rotterdam Study III). EasyQC software version 23.8 was used to clean and harmonize the data (23), and single‐nucleotide polymorphisms (SNPs) with a minor allele frequency <0.01 and imputation score <0.4 were removed. Meta‐analysis between the studies was performed using fixed‐effects inverse variance weighting using the Metal software package (24). A *P* value less than 5 × 10^−8^ was used to define genome‐wide significance, as is done routinely (25). The independent SNPs of interest for the AA were identified using the linkage disequilibrium clumping method (see MR methods below). A sensitivity analysis using the GCTA software COJO module was used to verify that the independent SNPs selected were also conditionally independent of each other (26). Genetic correlations and heritability were estimated using linkage disequilibrium score regression (27). See the Supplementary Methods for further details at https://onlinelibrary.wiley.com/doi/10.1002/art.42451.

### Downstream analyses

To identify genes responsible for genetic associations, colocalization was used to relate genetic signals found through GWAS to expression quantitative trait loci (QTLs) (28), which represent the genetic association between a locus and messenger RNA (mRNA) expression of a specific gene. Expression QTL data were examined from an online database (Genotype‐Tissue Expression [GTEx]) and from human cartilage tissue that is not readily available in GTEx (29). The presence of colocalization was defined as a posterior probability of >80% and considered suggestive if posterior probability was >60% (30). In addition, genetic loci were examined to see if they were predicted to be noncoding regulatory regions. Subsequently, immunohistochemistry staining in human knee osteochondral tissue was examined for any gene which colocalized in human cartilage. For further details, see the Supplementary Methods at https://onlinelibrary.wiley.com/doi/10.1002/art.42451.

### Mendelian randomization

MR was used to look for evidence of a causal effect between AA and hip OA using genetic instruments as proxies (15). Our AA GWAS meta‐analysis provided genetic instruments for AA. The hip OA genetic instruments were based on a GWAS of hospital‐diagnosed hip OA patients in the UK Biobank study. Bidirectional 2‐sample MR analysis was conducted using the inverse variance weighting method along with sensitivity analyses. These sensitivity analyses relax the assumption of no horizontal pleiotropy, assuming uncorrelated pleiotropy (MR–Egger regression), or relax the assumption about the number of invalid instruments (weighted median, simple mode, and weighted mode). Single‐SNP and leave‐one‐out analyses were performed to check that results were not driven by individual variants (17). Furthermore, we performed MR causal analysis using summary effect estimates (MR‐CAUSE) to examine for evidence of causality while considering the effects of both correlated and uncorrelated horizontal pleiotropy (31). The Strengthening the Reporting of Observational Studies in Epidemiology (STROBE) MR guidelines, which govern MR study reporting, provided a framework for this analysis (32). Further details are in the Supplementary Methods at https://onlinelibrary.wiley.com/doi/10.1002/art.42451.

### Data availability statement

The AA GWAS meta‐analysis summary statistics have been uploaded to the National Human Genome Research Institute–European Bioinformatics Institute GWAS Catalog (https://www.ebi.ac.uk/gwas/publications/36662418). The individual level data from this study will be available from the UK Biobank in a forthcoming data release. Users must be registered with the UK Biobank to access this resource (https://bbams.ndph.ox.ac.uk/ams).

## RESULTS

### Observational associations

To provide validity for our DXA measurement of AAs, the cross‐sectional association of an individual's AA measurement with clinical hip OA outcomes was examined in 40,337 individuals in our UK Biobank population (Supplementary Table 1, available at https://onlinelibrary.wiley.com/doi/10.1002/art.42451). The mean AA was 47.8° (range 31.8–115.0°), with a positively skewed distribution (Supplementary Figure 1) similar to a previous study (33). In both unadjusted and adjusted analyses, higher AA measurements were associated with hip pain (adjusted odds ratio [OR] 1.15 [95% confidence interval (95% CI) 1.11, 1.19]), radiographic hip OA grade ≥2 (adjusted OR 1.63 [95% CI 1.58, 1.67]), hospital‐diagnosed OA (adjusted OR 1.44 [95% CI 1.35, 1.54]), and subsequent total hip replacement (adjusted hazard ratio 1.45 [95% CI 1.33, 1.59]) (Table 1). Similar associations were seen when investigating cam morphology as a binary variable (Supplementary Table 2).

**Table 1:** Cross‐sectional and longitudinal associations between standardized alpha angle measurements and hip OA outcomes in the UK Biobank cohort*

Outcomes	Standardized alpha angle			
	Unadjusted		Adjusted†	
	OR or HR (95% CI)	*P*	OR or HR (95% CI)	*P*
Cross‐sectional analyses‡				
Hip pain	1.05 (1.01, 1.08)	6.98 × 10^−3^	1.15 (1.11, 1.19)	5.52 × 10^−14^
Radiographic hip OA grade ≥2	1.75 (1.70, 1.79)	1.00 × 10^−271^	1.63 (1.58, 1.67)	4.00 × 10^−271^
Radiographic hip OA grade ≥3	2.00 (1.92, 2.08)	3.00 × 10^−244^	1.91 (1.83, 2.00)	1.00 × 10^−174^
Radiographic hip OA grade 4	2.17 (2.00, 2.35)	2.10 × 10^−80^	2.10 (1.93, 2.30)	4.70 × 10^−62^
Hospital‐diagnosed OA	1.35 (1.27, 1.43)	1.70 × 10^−23^	1.44 (1.35, 1.54)	2.77 × 10^−28^
Longitudinal analysis§				
Total hip replacement	1.37 (1.27, 1.49)	1.18 × 10^−14^	1.45 (1.33, 1.59)	2.10 × 10^−17^

* Logistic regression was used to examine these associations except for the total hip replacement outcome, which was examined using Cox proportional hazards models. OA = osteoarthritis.

† Adjusted models include adjustment for age, sex, height, and weight.

‡ Cross‐sectional analyses are reported as the odds ratio (OR) (95% confidence interval [95% CI]) per SD increase in alpha angle.

§ Longitudinal analysis for total hip replacement is reported as the hazard ratio (HR) (95% CI) per SD increase in alpha angle.

### Alpha angle GWAS

The GWAS meta‐analysis comprised 44,214 total participants (Supplementary Figure 2 and Supplementary Table 3, available at https://onlinelibrary.wiley.com/doi/10.1002/art.42451). Eight statistically independent genome‐wide significant signals were observed (Table 2) (see Supplementary Table 4 for GCTA software COJO module results, Supplementary Figure 3 for a Manhattan plot of the analysis, and Supplementary Figure 4 for locus zoom plots, available at https://onlinelibrary.wiley.com/doi/10.1002/art.42451). The Q–Q plot showed some genetic inflation (λ = 1.08), which was expected given that the UK Biobank study provided most of the samples (Supplementary Figure 5). SNP trait heritability was modest (h^2^ = 0.10). After meta‐analysis, rs561578905 was the only genome‐wide significant SNP that was not present in the Rotterdam Study. To mitigate the effect of this, the SNP with the highest linkage disequilibrium (rs7302982, r^2^ = 0.77) was used instead for meta‐analysis of the Rotterdam Study data. Three SNPs showed weak evidence of heterogeneity (rs7571789, I^2^ = 53%, heterogeneity *P* = 0.09; rs10478422, I^2^ = 33%, *P* = 0.21; and rs561578905, I^2^ = 25%, *P* = 0.26) (Supplementary Figure 6).

**Table 2:** Top independent SNPs associated with alpha angle from a GWAS meta‐analysis of data from the UK Biobank study and Rotterdam Study*

	Closest gene	Chromosome	Base position	Effect allele	Non‐effect allele	Effect allele frequency	β	*P*
rs7571789	*TGFA*	2	70714793	T	C	0.48	0.04	7.52 × 10^−09^
rs10478422	*TNFAIP8*	5	118747441	T	C	0.30	0.04	9.64 × 10^−10^
rs1048584	*TFB1M*	6	155578599	A	T	0.39	–0.04	7.67 × 10^−09^
rs62578126	*LMX1B*	9	129375338	T	C	0.37	–0.04	9.00 × 10^−09^
rs10787959	*GRK5*	10	121131313	A	G	0.28	–0.04	1.08 × 10^−08^
rs561578905†	*SOX5*	12	24206118	A	C	0.27	0.05	3.37 × 10^−08^
rs146939415	*CYP19A1*	15	51522210	C	G	0.01	0.17	2.47 × 10^−08^
rs4911180	*UQCC1*	20	33972948	A	G	0.63	–0.04	1.25 × 10^−11^

* Results presented are from the fixed‐effects meta‐analysis after linkage disequilibrium clumping. Only single‐nucleotide polymorphisms (SNPs) with minor allele frequency ≥0.01 and *P* < 5 × 10^−8^ are listed. The UK Biobank genome‐wide association study (GWAS) was adjusted for age, sex, genetic chip, and 20 principal components, and the Rotterdam Study GWAS was adjusted for age, sex, and 4 principal components.

† The SNP rs561578905 was not available in the Rotterdam Study, and rs7302982 (r^2^ = 0.77) was used instead for meta‐analysis of the Rotterdam Study data.

The top 8 SNPs showed the same direction of effect in a GWAS of cam morphology as a binary variable (assessed in 38,173 individuals from the UK Biobank with an AA ≥60°), albeit with *P* values that did not reach our genome‐wide significance threshold (Supplementary Table 5). The closest gene to each independent SNP associated with AA was initially used to label the loci, namely *TGFA*, *TNFAIP8*, *TFB1M‐TIAM2*, *LMX1B*, *GRK5*, *SOX5*, *CYP19A1*, and *UQCC1* (Table 2). *TGFA*, *LMX1B*, *SOX5*, *CYP19A1*, and *UQCC1‐GDF5* loci have previously been associated with OA (34). The *LMX1B* locus shared the same lead SNP for AA and OA association, whereas *SOX5*, *TGFA*, and *UQCC1‐GDF5* AA‐associated SNPs were in moderate‐to‐high linkage disequilibrium (r^2^ = 0.30, r^2^ = 0.65, and r^2^ = 0.79, respectively) and *CYP19AI* showed only very weak linkage disequilibrium (r^2^ = 0.06) with their OA‐associated equivalents.

### Downstream analysis of AA associations

In expression QTL analyses, AA genetic association signals for rs10478422, rs62578126, and rs1048584, respectively, colocalized with mRNA expression of *TNFAIP8* in cultured fibroblasts (504 samples; posterior probability of 97%)*, LMX1B* in adipose tissue (663 samples; posterior probability of 96%), and *CLDN20*/*RP11‐477D19*/*TFB1M* in cultured fibroblasts (504 samples; posterior probabilities of 87%, 90%, and 66%, respectively), and these genes underlie the genetic associations observed (Supplementary Table 6, https://onlinelibrary.wiley.com/doi/10.1002/art.42451). In further expression QTL analyses based on human cartilage samples, the AA genetic association signal at the *TNFAIP8* locus colocalized with the *TNFAIP8* mRNA expression in highly degraded human cartilage (115 samples; posterior probability of 97%) (Supplementary Figure 7). No other SNPs showed evidence of colocalization with expression QTL data from less degraded or highly degraded cartilage (Supplementary Table 7). We evaluated the likely effect of identified SNPs on DNA–protein interactions using the RegulomeDB database, which predicts the impact of the base change on DNA binding of transcription regulators. The rs7571789 (*TGFA*), rs6595186 (*TNFAIP8)*, rs62578126 (*LMX1B)*, rs561578905 (*SOX5)*, and rs246939415 (*CYP19A1)* SNPs were all predicted to affect enhancer or promotor activity (Supplementary Table 8), suggesting they may be directly responsible for the genetic association observed.

**Figure 2:**
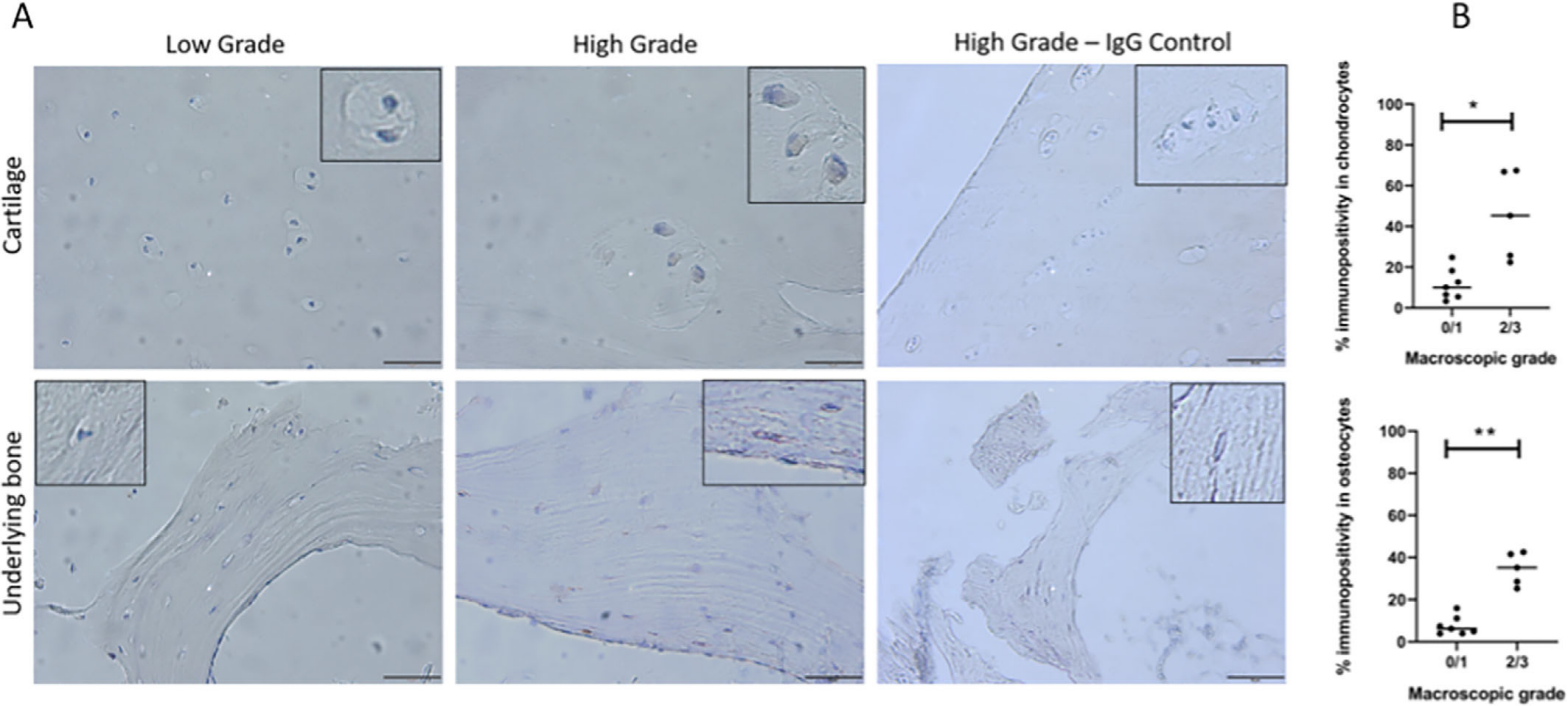
Immunohistochemistry localization of tumor necrosis factor–induced protein 8 (TNFAIP‐8) within human osteochondral tissues. **A**, Immunohistochemistry staining in middle zone cartilage and underlying bone identifying TNFAIP‐8 in chondrocytes and osteocytes, particularly in highly degraded (high grade) tissues. IgG controls were negative. Scale bar = 50 μm. **B**, Percentage TNFAIP‐8 immunopositivity in chondrocytes (top) and osteocytes (bottom). Symbols represent individual patients; horizontal lines show the median. * = *P* = 5.1 × 10^−3^; ** = *P* = 2.5 × 10^−3^. Color figure can be viewed in the online issue, which is available at http://onlinelibrary.wiley.com/doi/10.1002/art.42451/abstract.

Given the finding that the AA genetic association signal at the *TNFAIP8* locus colocalized with *TNFAIP8* expression in highly degraded human cartilage, we used immunohistochemistry to further explore *TNFAIP8* expression in human knee cartilage and underlying bone in 4 patient samples. Tumor necrosis factor–induced protein 8 (TNFAIP‐8) immunopositivity was localized to chondrocytes and osteocytes within the osteochondral tissue samples (Figure 2A). An increase in percentage immunopositivity in chondrocytes (*P* = 5.1 × 10^−3^) and osteocytes (*P* = 2.5 × 10^−3^) was seen in highly degraded compared to less degraded tissues (Figure 2B).

### Genetic correlations

The interstudy AA genetic correlation was reasonable (r_g_ = 0.57 [95% CI 0.05, 1.09]), but the estimate was unreliable due to the small sample size of the Rotterdam Study GWAS (Supplementary Table 9, https://onlinelibrary.wiley.com/doi/10.1002/art.42451). There was a moderate genetic correlation between AA and hip OA (r_g_ = 0.26 [95% CI 0.10, 0.43]) and minimum joint space width (r_g_ = –0.31 [95% CI –0.46, –0.15]), with an inverse relationship observed in the AA and minimum joint space width correlation as expected. There was no or very limited evidence of a genetic correlation with hip pain, height, body mass index, bone mineral density, or fracture occurrence (Supplementary Table 9).

### Mendelian randomization

**Figure 3:**
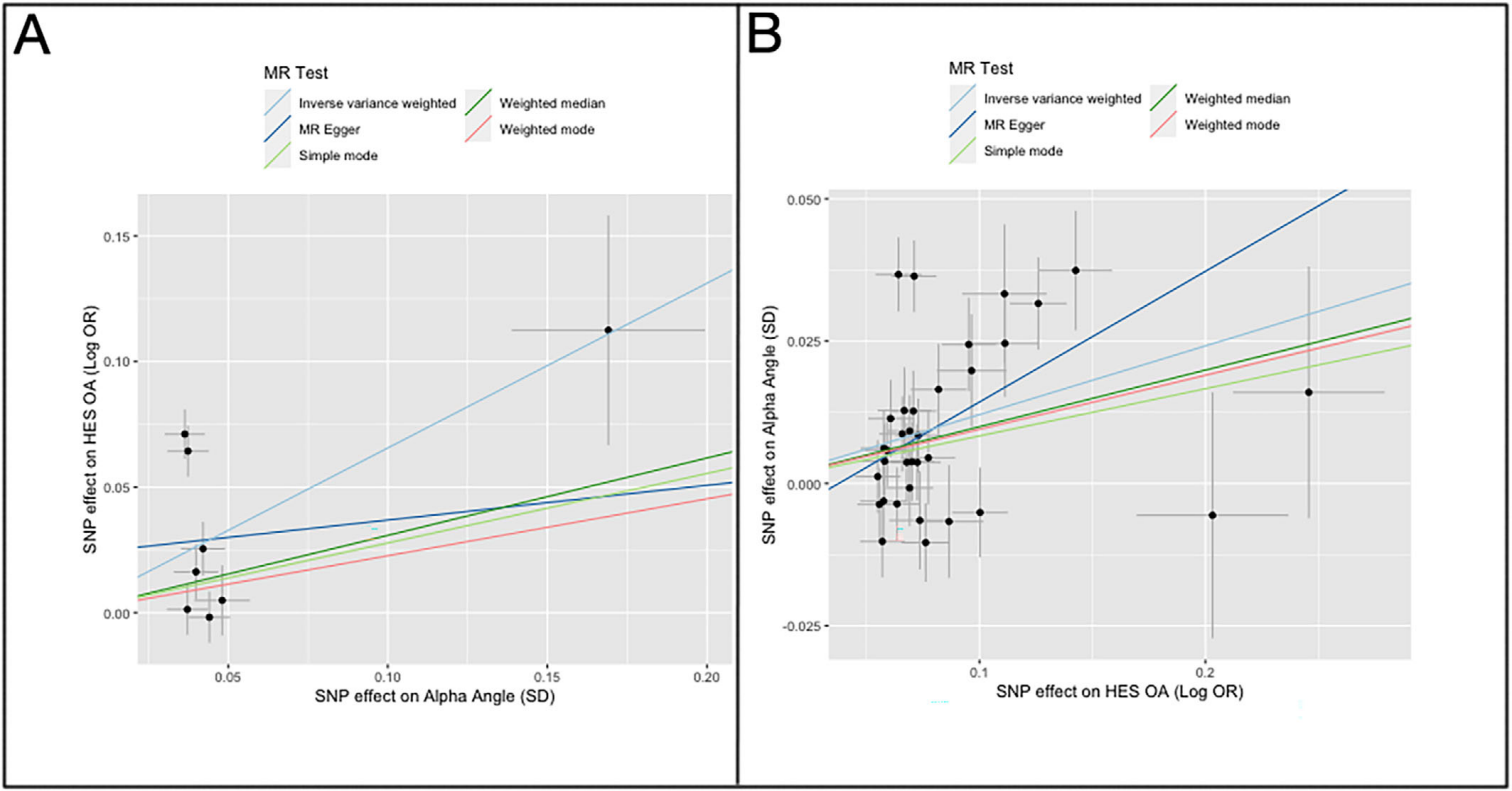
Bidirectional Mendelian randomization (MR) results comparing the causal effects between alpha angle and hospital‐diagnosed hip osteoarthritis (OA) in the UK Biobank study. **A**, MR analyses using 8 single‐nucleotide polymorphisms (SNPs) as genetic instruments for alpha angle as the exposure (x‐axis) and risk of hospital‐diagnosed hip osteoarthritis (hospital episode statistics [HES] OA) as the outcome (y‐axis). **B**, MR analyses using 34 SNP genetic instruments for risk of hip OA as the exposure (x‐axis) and alpha angle as the outcome (y‐axis). Symbols with horizontal and vertical lines represent individual SNPs and 95% confidence intervals. OR = odds ratio. Color figure can be viewed in the online issue, which is available at http://onlinelibrary.wiley.com/doi/10.1002/art.42451/abstract.

We subsequently performed an MR analysis of AA versus hip OA risk using the 8 SNPs identified above as our genetic instrument for AA (Table 2). The mean F statistic for our AA genetic instrument was 31.5, indicating acceptable instrument strength. Inverse variance weighting analysis, the primary MR test statistic, provided weak evidence of an effect of increasing AA on hip OA risk (OR per SD increase in AA 1.84 [95% CI 1.14, 2.96]) (Table 3). Additional MR sensitivity analyses, including MR–Egger regression, showed no evidence of a causal effect of AA on hip OA risk (Figure 3). Inverse variance weighting and MR–Egger regression Q statistics were 56.1 and 54.4, respectively, indicating heterogeneity and possible pleiotropy. The individual AA‐associated SNP effects for hip OA are shown in Supplementary Table 5 at https://onlinelibrary.wiley.com/doi/10.1002/art.42451. Further sensitivity analyses, in the form of leave‐one‐out and single‐SNP analyses were performed; however, similar effect estimates were observed (Supplementary Figures 8 and 9), suggesting no single SNP was responsible for the heterogeneity.

**Table 3:** Bidirectional Mendelian randomization results comparing the causal effects between alpha angle and hip OA*

MR method	Exposure alpha angle; outcome hip OA		Exposure hip OA; outcome alpha angle	
	Odds ratio (95% CI)†	*P*	β (95% CI)‡	*P*
Inverse variance weighting	1.84 (1.14, 2.96)	0.01	0.09 (0.04, 0.13)	4.58 × 10^−5^
MR–Egger regression	1.22 (0.18, 8.37)§	0.84	0.15 (0.01, 0.30)¶	0.05
Weighted median	1.22 (0.93, 1.59)	0.16	0.08 (0.04, 0.12)	7.77 × 10^−5^
Simple mode	1.33 (0.91, 1.93)	0.16	0.10 (0.00, 0.19)	0.05
Weighted mode	1.18 (0.92, 1.51)	0.27	0.12 (0.02, 0.21)	0.02

* Risk of hip osteoarthritis (OA) derived from UK Biobank genome‐wide association study of hospital‐diagnosed hip OA. 95% CI = 95% confidence interval.

† Odds ratio of hip OA per SD increase in alpha angle.

‡ Beta value per doubling in odds of hip OA.

§ Mendelian randomization (MR)–Egger regression intercept = 0.02, *P* = 0.68.

¶ MR–Egger regression intercept = –0.01, *P* = 0.34.

For MR analyses in the opposite direction, the hip OA GWAS (323,948 participants) identified 34 independent SNPs, with a mean F statistic of 45.0, indicating acceptable instrument strength (Supplementary Table 10). In inverse variance weighting analyses, there was reasonably strong evidence of a causal effect of increasing hip OA risk on AA (β = 0.09 [95% CI 0.04, 0.13], where β is SD change in AA per doubling in odds of hip OA) (Table 3). Sensitivity analyses were broadly in agreement (Figure 3). Inverse variance weighting and MR–Egger regression Q statistics were 97.6 and 94.8, respectively, indicating heterogeneity and possible pleiotropy.

MR‐CAUSE analyses, which use whole GWAS summary statistics, were performed to examine for causal effects and for correlated pleiotropy, whereby 2 traits are related to each other as a result of shared pathways (31). For AA versus hip OA, there was only weak evidence that the causal model (model 2) performed better than the null model (model 1) (expected log pointwise predictive density [ELPD] = –3.80, *P* = 0.07, where ELPD ≤0 suggests model 2 fits the data better than model 1) (Supplementary Table 11). For hip OA versus AA, there was stronger evidence that the causal model (model 2) performed better than the null model (model 1) (ELPD = –7.12, *P* = 0.03) and better than the model assuming correlated pleiotropy (sharing model) (ELPD = –3.65, *P* = 0.02).

## DISCUSSION

This is the first GWAS meta‐analysis of AA, which identified 8 loci and indicated a heritable component of 10%. *TGFA*, *TNFAIP8*, *CLDN20*/*RP11‐477D19*/*TFB1M*, *LMX1B*, *GRK5*, *SOX5*, *CYP19A1*, and *UQCC1* were implicated in increasing AA, with *TNFAIP8* showing the strongest gene–SNP relationship. Despite strong evidence of observational associations, bidirectional 2‐sample MR analyses provided limited evidence of a causal association between increasing AA and the development of hip OA, but rather showed greater evidence that a genetic predisposition to hip OA causes an increase in AA, as measured by DXA in this cohort.

Among these 8 loci, the AA genetic association signal at the *TNFAIP8* locus colocalized with *TNFAIP8* mRNA expression in chondrocytes obtained from highly degraded cartilage, but not healthy cartilage, of the same individual, indicating *TNFAIP8* is the likely effector gene for this GWAS locus. This suggestion that *TNFAIP8* is preferentially expressed in degraded cartilage was further explored by subsequent immunohistochemistry staining, which showed greater expression of *TNFAIP8* in chondrocytes and osteocytes from degraded joint tissue. TNFAIP‐8 is a tumor necrosis factor–binding protein forming part of inflammatory, catabolic, and neurosensitization pathways during the pathogenesis of OA and is also involved in cell apoptosis (35). Inflammatory changes are well recognised in hip OA (36). Our findings raise the possibility that TNFAIP‐8 expression in osteocytes and chondrocytes contributes to hip shape modeling in the setting of OA; however, further work is required to establish the mechanisms involved. How hip shape and an individual's AA change over time is not well understood, and it could be that the observed shape variation arises in later life as part of the hip OA process, distinct from cam morphology caused by altered shape development in adolescence (9).

AA genetic association signals at the rs62578126 and rs1048584 loci showed evidence of colocalization with mRNA expression of *LMX1B* and *CLDN20*/*RP11‐477D19*/*TFB1M* in adipose tissue and fibroblasts, respectively. Although the same colocalization was not seen with mRNA expression in chondrocytes, this provides some evidence that these genes may be responsible for the genetic associations found with AA. These discrepancies might be explained by adipose and fibroblast cells being the effector cells in this disease process or that the chondrocyte analysis had less power. *LMX1B* is the gene responsible for nail–patella syndrome, which involves poorly developed nails and patella and multiple limb malformations, and when knocked out in mice, is associated with abnormal ventral limb development (37). *TFB1M* is important in preventing oxidative stress in mitochondria in the context of OA (38). *CLDN20* is from the claudin family, which is known to regulate osteoblast activity (39). *RP11‐477D19* was also identified through colocalization, although little is known about its function.

The other SNPs provided no specific evidence of a causal gene through expression QTL or colocalization analyses. Nonetheless, many of the closest genes identified have previously been implicated in limb development and in OA. An intronic variant of *UQCC1* has been associated with developmental dysplasia of the hip in a Han Chinese population (40). Interestingly, our *UQCC1* SNP (rs4911180) was in high linkage disequilibrium with the lead *GDF5* OA‐associated SNP (r^2^ = 0.79) from a previous GWAS (34). The *UQCC1–GDF5* locus has been implicated in abnormal limb development and in OA, with both genes commonly expressed in chondrocytes (41, 42, 43). *TGFA* (rs7571789) is a growth factor that is expressed in developing limbs in chicks (44) and is important in the development of OA (45). *SOX5* (rs561578905) has previously been shown to be critical in joint morphogenesis through its action on growth plate and articular chondrocytes (46). *CYP19A1* (rs146939415) has been associated with large joint OA and is thought to act via aromatase inhibition (47). Finally, *GRK5* is thought to regulate cartilage degradation and might be a possible therapeutic target for OA (48).

The 8 independent AA‐associated SNPs had acceptable instrument strength when combined in subsequent MR analyses, suggesting they were good genetic proxies for AA. However, there was only weak evidence of a causal link between increased AA and hip OA. Interestingly, our bidirectional MR study provided stronger evidence that a genetic predisposition for hip OA causes an increased AA, suggesting that the morphologic features identified in this cohort may develop as part of, or in parallel to, the hip OA process. Modeling changes are recognized by the Croft scoring system in late‐stage hip OA (12); however, we are not aware of any previous reports describing cam‐like morphology as a specific feature of hip OA. That said, in the same set of DXA images, we recently found that changes in hip shape suggestive of cam morphology were associated with more severe forms of hip OA (11).

Although the evidence for a causal effect of a genetic predisposition to hip OA on AA was somewhat stronger than that of AA on risk of hip OA, it should be noted that our genetic instrument for hip OA was stronger than that for AA, reflecting a greater number of SNPs, so caution needs to be exercised in comparing these effects. Moreover, given that our cohort was 44–82 years of age, variation in AA may largely have reflected modeling changes that are part of the hip OA disease process as opposed to cam morphology developed in earlier life. Alternatively, rather than bidirectional causal effects, it may be that our findings reflect common genetic pathways involved in the development of increased AA and hip OA, causing them to develop in parallel rather than as a consequence of each other. Though MR‐CAUSE analysis favored a causal over a shared model, this was only supported by weak evidence, and given the disparity in instrument strength between the 2 traits, it is difficult to reach any firm conclusions. Nevertheless, to the extent that a causal effect of a genetic predisposition of hip OA on AA and/or a causal effect of shared genetic pathways contribute to associations between an increased AA and hip OA, our results suggest that hip OA should not necessarily be attributed to cam morphology, especially in individuals in whom increased AA and hip OA might coexist. This has implications when considering hip shape remodeling surgery in an older adult population.

In this study, AA as a continuous measure was used as a proxy for cam morphology. There are several limitations to this approach. For example, measuring AA on anteroposterior images can be partially out of plane to the cam lesion, leading to an underestimation of size. Furthermore, AA measurements in the UK Biobank study were obtained from DXA images rather than x‐ray images, which might lead to discrepancies between these 2 modalities. However, despite this potential discrepancy, our observational analyses suggest we measured a clinically relevant shape signal from DXA images. Moreover, any failure to detect cam lesions that were present would tend to reduce associations to the null rather than produce biased estimates. When measuring AA in a population in later life there is the possibility that AA measurements capture osteophytes or other hip shape features of OA. However, we rigorously excluded osteophytes, and if these were included in our measurements, we might have expected to see a stronger causal relationship between AA and hip OA. The newer generation of DXA scanner used in the UK Biobank study has a similar resolution to plain radiographs, and some authors report comparable ability to detect osteophytes (49).

Although we used AA as a continuous measure to optimize statistical power, this method has less clinical relevance than dichotomizing into the presence or absence of cam morphology based on a predefined cutoff (2, 22). That said, we found a similar observational relationship between AA and cam morphology and hip OA outcomes irrespective of whether we used a continuous (AA) or binary (cam morphology) measure. Moreover, sensitivity analyses based on a binary AA variable showed similar but underpowered GWAS results. Further work is needed to recruit hip imaging cohorts that are closer to that of the UK Biobank in terms of scale and phenotyping to allow for further replication of our results and to extend our findings to more ethnically diverse populations. Finally, as with any MR study, several assumptions needed to be made. The relevance assumption is satisfied by our ample F statistics, but the independence and exclusion restriction assumptions are harder to test (15). Several sensitivity analyses were performed to examine for possible pleiotropy that suggested pleiotropy was present.

In conclusion, using a novel GWAS meta‐analysis of the AA, our study suggests that causal relationships between AA and hip OA, and particularly a genetic predisposition for hip OA and AA, contribute to observational associations between hip OA and AA in an older population. Changes in AA as a consequence of hip OA development may involve up‐regulation of inflammatory and catabolic pathways, given our observation that *TNFAIP8*, one of the top AA‐associated loci, was preferentially expressed in degraded human articular cartilage and bone. Further studies are necessary to explore the contribution of increased AA to clinical consequence of hip OA and to determine whether targeting the underlying molecular mechanisms might prove useful in ameliorating hip OA disease development and progression.

## AUTHOR CONTRIBUTIONS

All authors were involved in drafting the article or revising it critically for important intellectual content, and all authors approved the final version to be published. Dr. Faber had full access to all of the data in the study and takes responsibility for the integrity of the data and the accuracy of the data analysis.

### Study conception and design

Faber, Aspden, Harvey, Le Maitre, Wilkinson, van Meurs, Zeggini, Cootes, Lindner, Kemp, Davey Smith, Tobias.

### Acquisition of data

Faber, Aspden, Harvey, Le Maitre, Wilkinson, van Meurs, Zeggini, Cootes, Lindner, Kemp, Davey Smith, Tobias.

### Analysis and interpretation of data

Faber, Frysz, Hartley, Ebsim, Boer, Saunders, Gregory, Southam, Giles.

## Supporting information


Disclosure Form

**Appendix S1:** Supporting Information

**Supplementary Figure 1** Alpha angle distribution in UK Biobank
**Supplementary Figure 2**. A flow chart of the populations used in this study.
**Supplementary Figure 3**. A Manhattan Plot describing the alpha angle GWAS meta‐analysis. The closest genes label the independent genetic loci that meet genome‐wide significance.
**Supplementary Figure 4a**. Locus zoom plot rs7571789 (*TGFA*)
**Supplementary Figure 4b**. Locus zoom plot rs455991 (*TNFAIP8*)
**Supplementary Figure 4c**. Locus zoom plot rs1048584 (*TIAM2‐TFB1M*)
**Supplementary Figure 4d**. Locus zoom plot rs62578126 (*LMX1B*)
**Supplementary Figure 4e**. Locus zoom plot rs10787959 (*GRK5*)
**Supplementary Figure 4f**. Locus zoom plot rs146939415 (*CYP19A1*)
**Supplementary Figure 4g**. Locus zoom plot rs4911180 (*UQCC1*)
**Supplementary Figure 4h**. Locus zoom plot rs561578905 (*SOX5*)
**Supplementary Figure 5**. QQ plot for alpha angle GWAS meta‐analysis
**Supplementary Figure 6**. A Forest Plot for each independent SNP.
The SNP effects from each cohort are displayed. The exponentiated beta is displayed to aid visualisation. The heterogeneity statistic (I^2^) was zero for all SNPs apart from rs7571789 (I^2^ = 53, P‐value = 0.09), rs10478422 (I^2^ = 33, P = 0.21) and rs561578905 (I^2^ = 25, P = 0.26). RS ‐ Rotterdam Study, UKB ‐ UK Biobank, Meta ‐ Meta‐analysis.
**Supplementary Figure 7**. A colocalisation plot for *TNFAIP8* expression in highly degraded human cartilage.
**Supplementary Figure 8a**. Leave one out analysis comparing alpha angles effect on hip osteoarthritis.
**Supplementary Figure 8b**. Leave one out analysis comparing hip osteoarthritis effect on alpha angle.
**Supplementary Figure 9a**. Single SNP analysis of alpha angles effect on hip osteoarthritis.
**Supplementary Figure 9b**. Single SNP analysis of hip osteoarthritis effect on alpha angle.

**Supplementary Table 1** Descriptives for the UK Biobank individuals included in this study.
**Supplementary Table 2**. Observational associations between cam morphology and clinical outcomes
**Supplementary Table 3**. Alpha angle GWAS population
**Supplementary Table 4**. COJO results for AA meta‐analysis
**Supplementary Table 5**. Look up of AA SNPs in a UKB Cam GWAS and HOA GWAS
**Supplementary Table 6**. GTEx look up results for the independent alpha angle SNPs
**Supplementary Table 7**. Colocalisation results between each independent AA loci and eQTL data from human cartilage
**Supplementary Table 8**. RegulomeDB results for each independent SNP.
**Supplementary Table 9**. Linkage disequilibrium score regression between AA, UKB HES OA and Meta‐analysis of HOA
**Supplementary Table 10**. Genetic instruments for Hospital diagnosed hip OA obtained from a GWAS of HES OA in UKB, followed by LD clumping after palindromic SNPs were removed.
**Supplementary Table 11**. MR‐CAUSE analysis
