# Supplementary material for: A Genome‐Wide Association Study Meta‐Analysis of Alpha Angle Suggests Cam‐Type Morphology May Be a Specific Feature of Hip Osteoarthritis in Older Adults
Source: Arthritis Rheumatol. 2023 Apr 9;75(6):900–9. doi: 10.1002/art.42451 (PMC10374163; doi:10.1002/art.42451)
Supplement: Supplementary file 3 — Supplementary Figure 1 Alpha angle distribution in UK Biobank Supplementary Figure 2. A flow chart of the populations used in this study. Supplementary Figure 3. A Manhattan Plot describing the alpha angle GWAS meta‐analysis. The closest genes label the independent genetic loci that meet genome‐wide significance. Supplementary Figure 4a. Locus zoom plot rs7571789 (TGFA) Supplementary Figure 4b. Locus zoom plot rs455991 (TNFAIP8) Supplementary Figure 4c. Locus zoom plot rs1048584 (TIAM2‐TFB1M) Supplementary Figure 4d. Locus zoom plot rs62578126 (LMX1B) Supplementary Figure 4e. Locus zoom plot rs10787959 (GRK5) Supplementary Figure 4f. Locus zoom plot rs146939415 (CYP19A1) Supplementary Figure 4g. Locus zoom plot rs4911180 (UQCC1) Supplementary Figure 4h. Locus zoom plot rs561578905 (SOX5) Supplementary Figure 5. QQ plot for alpha angle GWAS meta‐analysis Supplementary Figure 6. A Forest Plot for each independent SNP. The SNP effects from each cohort are displayed. The exponentiated beta is displayed to aid visualisation. The heterogeneity statistic (I2) was zero for all SNPs apart from rs7571789 (I2 = 53, P‐value = 0.09), rs10478422 (I2 = 33, P = 0.21) and rs561578905 (I2 = 25, P = 0.26). RS ‐ Rotterdam Study, UKB ‐ UK Biobank, Meta ‐ Meta‐analysis. Supplementary Figure 7. A colocalisation plot for TNFAIP8 expression in highly degraded human cartilage. Supplementary Figure 8a. Leave one out analysis comparing alpha angles effect on hip osteoarthritis. Supplementary Figure 8b. Leave one out analysis comparing hip osteoarthritis effect on alpha angle. Supplementary Figure 9a. Single SNP analysis of alpha angles effect on hip osteoarthritis. Supplementary Figure 9b. Single SNP analysis of hip osteoarthritis effect on alpha angle. [file ART-75-900-s003.docx]

Supplementary Figures:

Supplementary Figure 1: Alpha angle distribution in UK Biobank

DXA and AA measure available in UKB (n=40,337). Observational associations done in this population

Those that have an AA measure and pass genetic quality control in UK Biobank

(n = 38,173)

Alpha Angle GWAS meta-analysis (n=44,214)

Individuals who fail genetic quality control in UK Biobank (n=2,164)

Those that have an AA measure and pass genetic quality control in the Rotterdam Studies (n = 6,041)

Supplementary Figure 2. A flow chart of the populations used in this study.


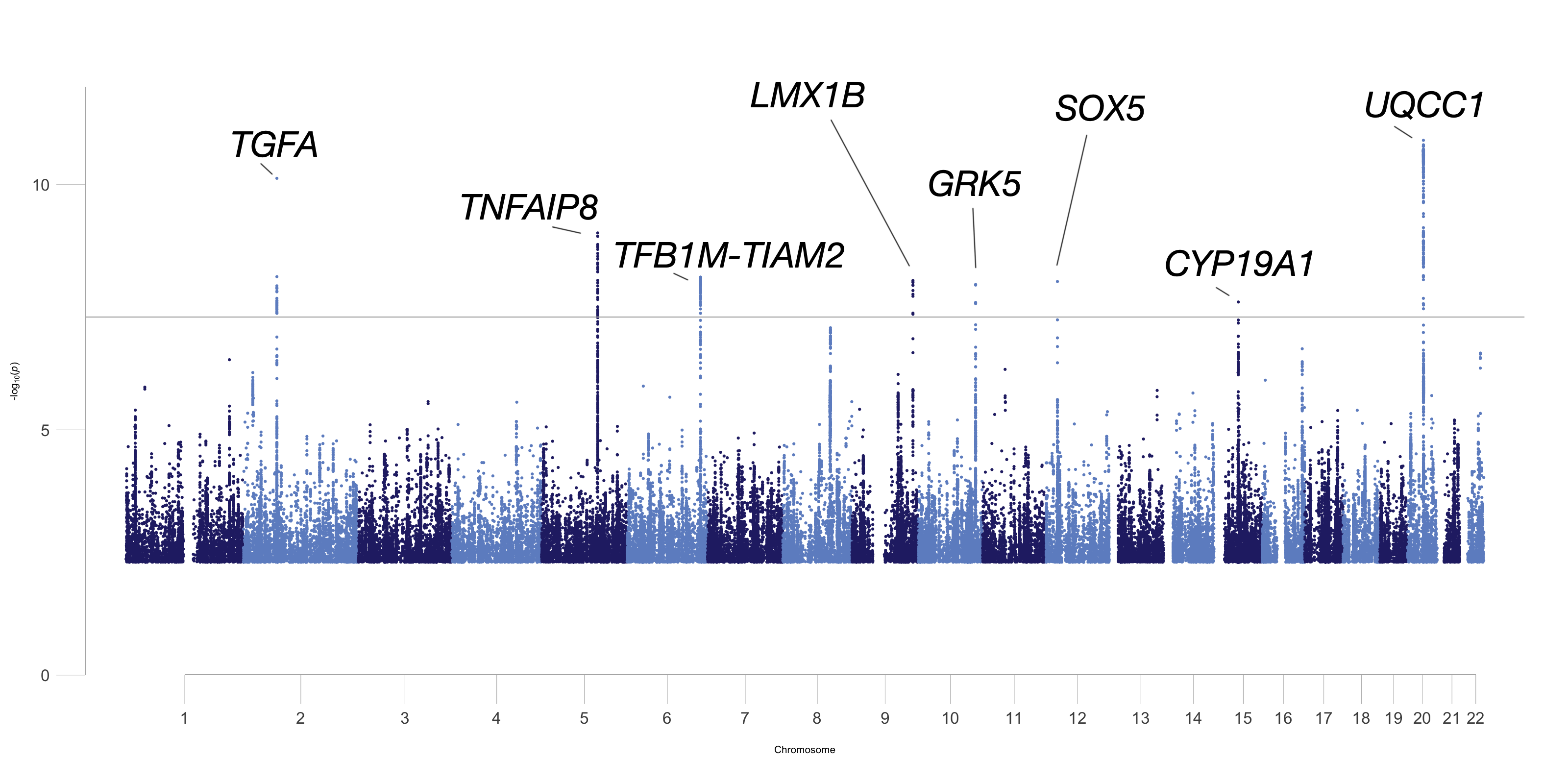


Supplementary Figure 3. A Manhattan Plot describing the alpha angle GWAS meta-analysis. The closest genes label the independent genetic loci that meet genome-wide significance.


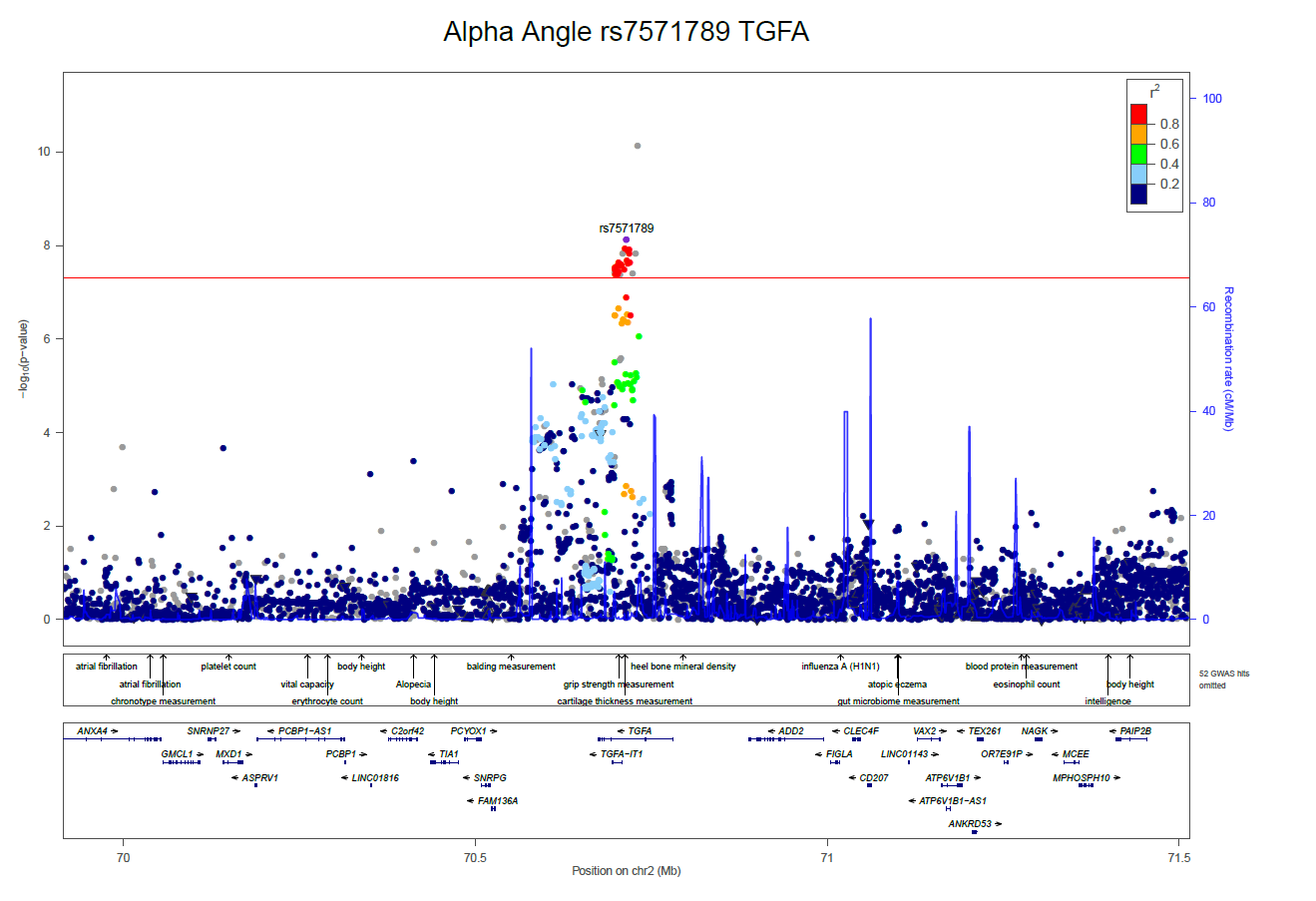


Supplementary Figure 4a. Locus zoom plot rs7571789 (*TGFA*)


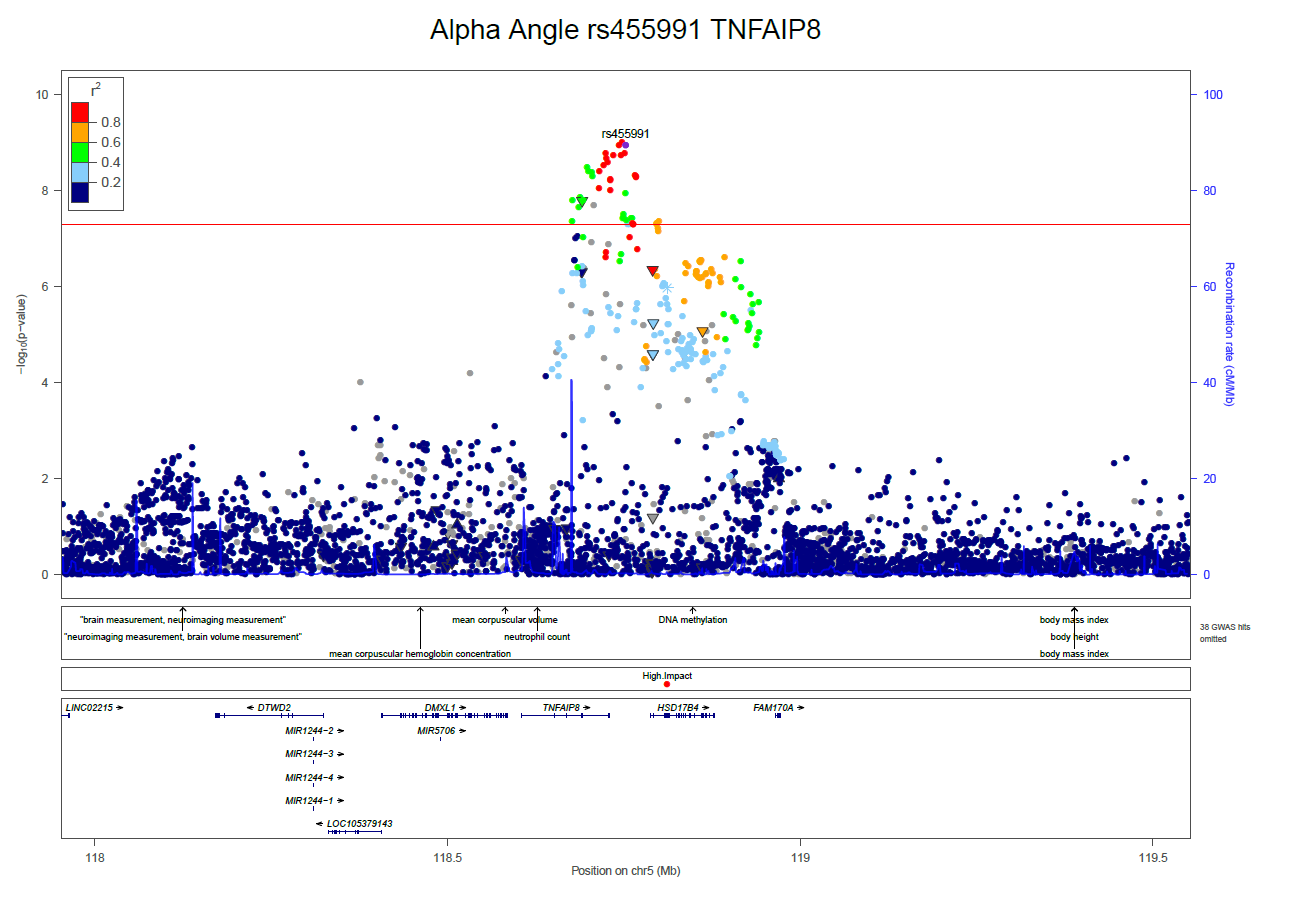


Supplementary Figure 4b. Locus zoom plot rs455991 (*TNFAIP8*)


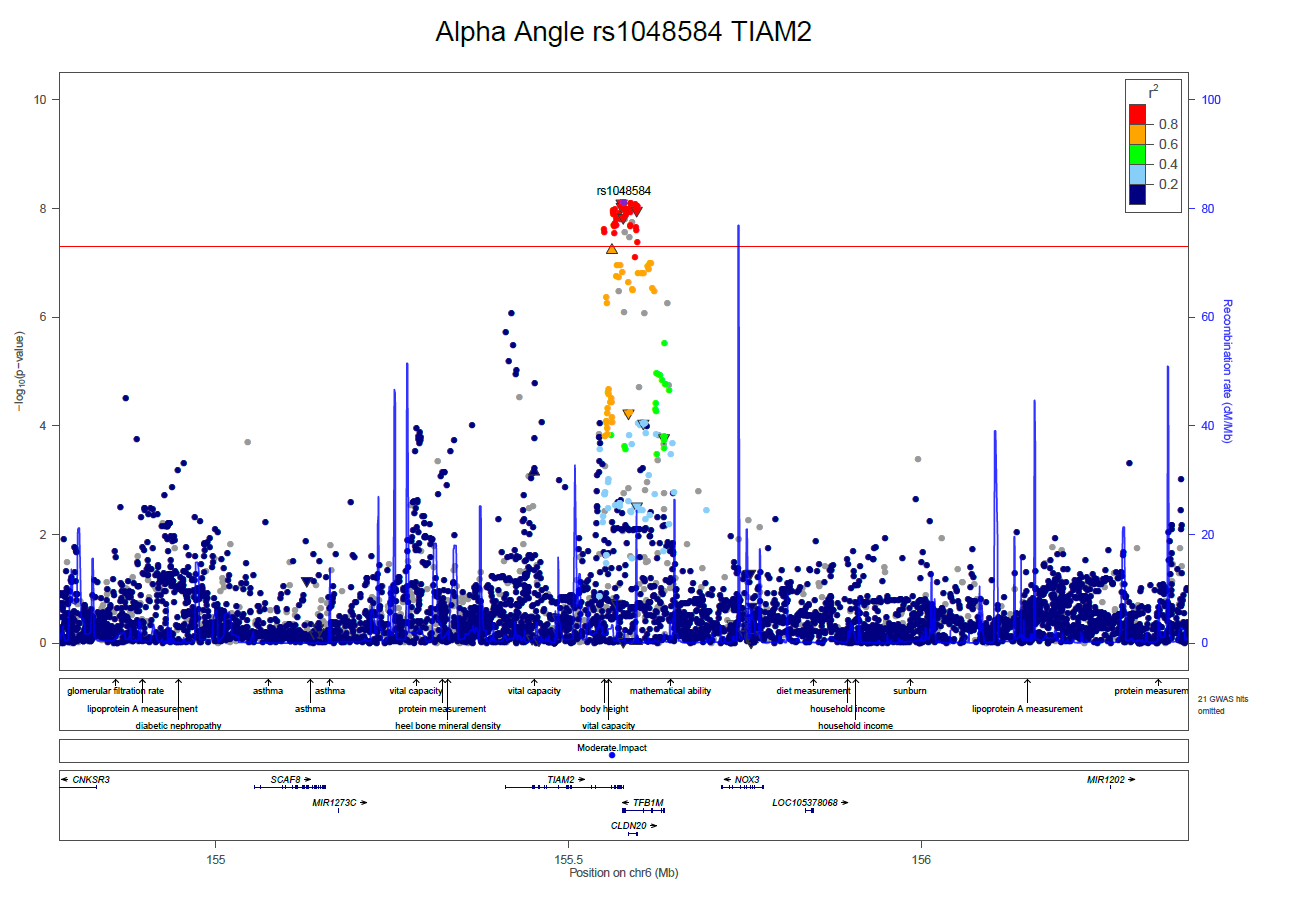


Supplementary Figure 4c. Locus zoom plot rs1048584 (*TIAM2-TFB1M*)


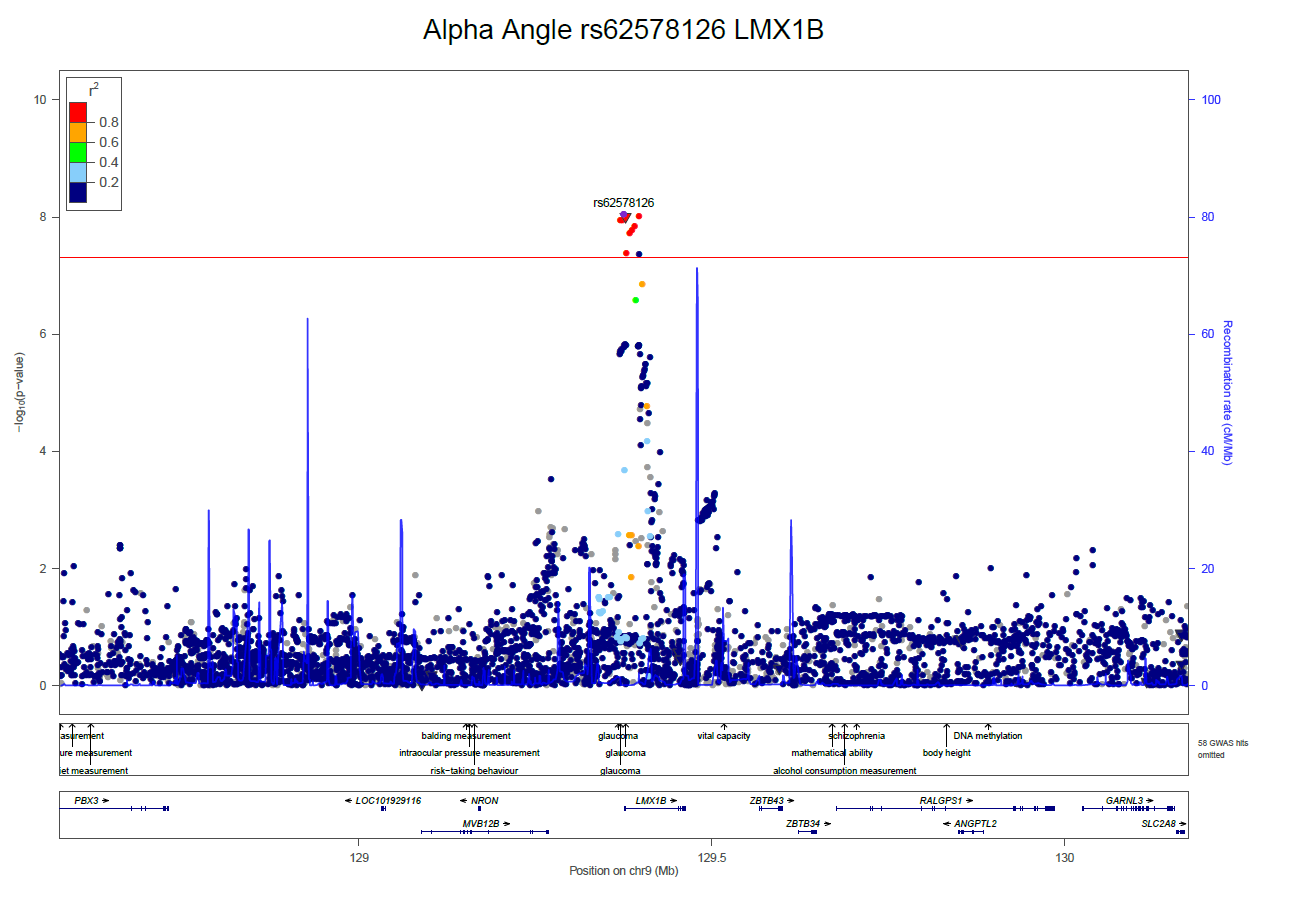


Supplementary Figure 4d. Locus zoom plot rs62578126 (*LMX1B*)


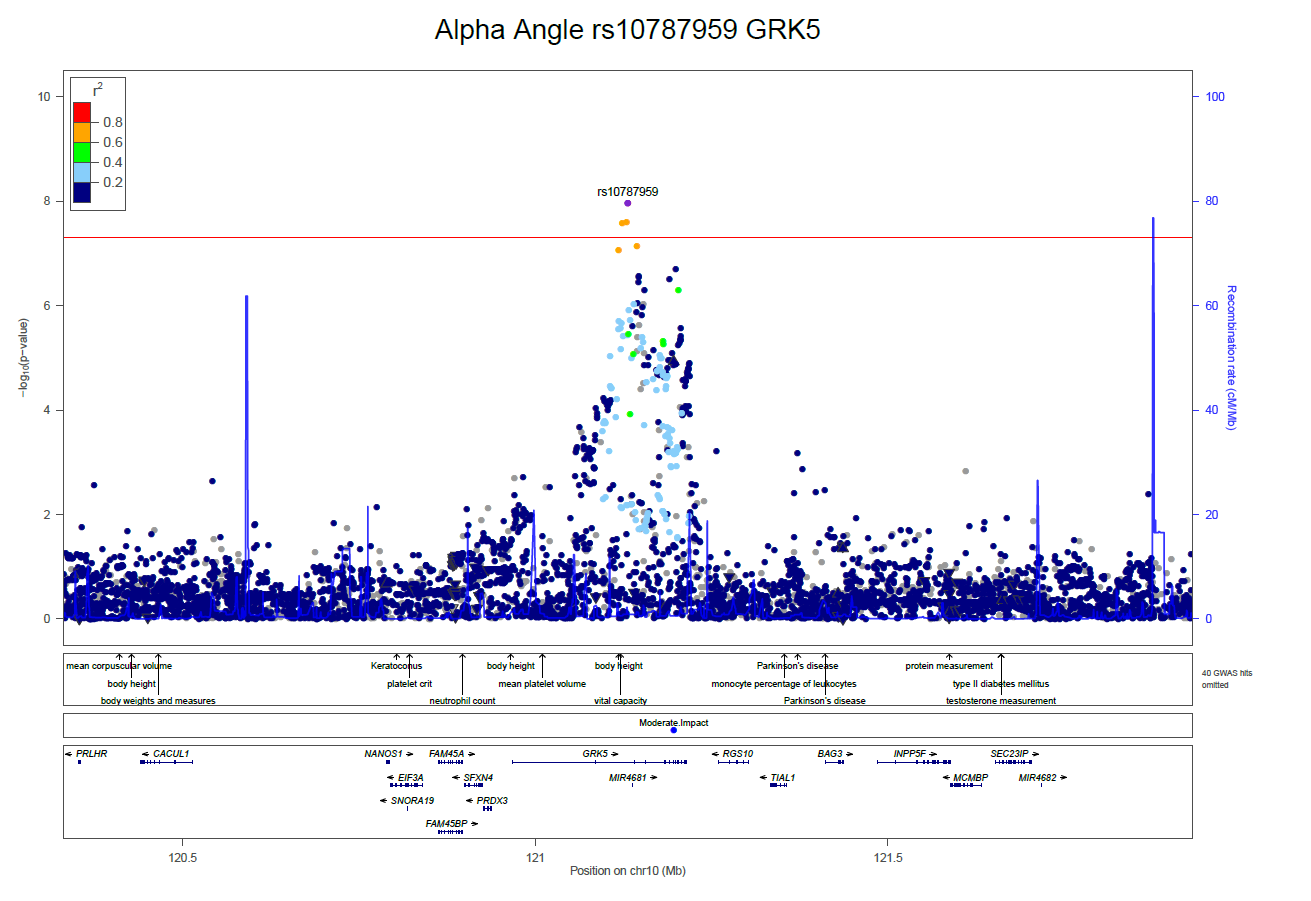


Supplementary Figure 4e. Locus zoom plot rs10787959 (*GRK5*)


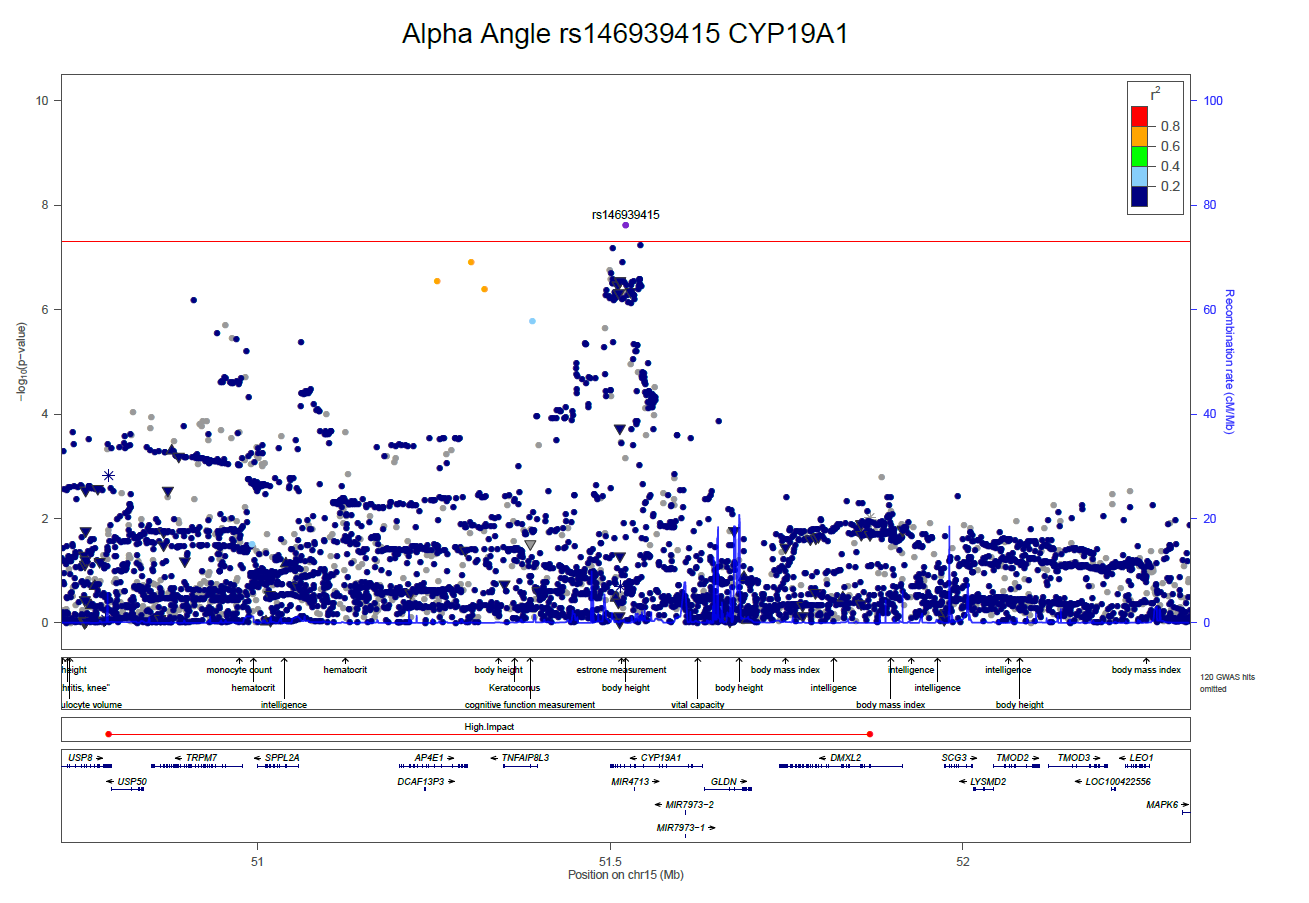


Supplementary Figure 4f. Locus zoom plot rs146939415 (*CYP19A1*)


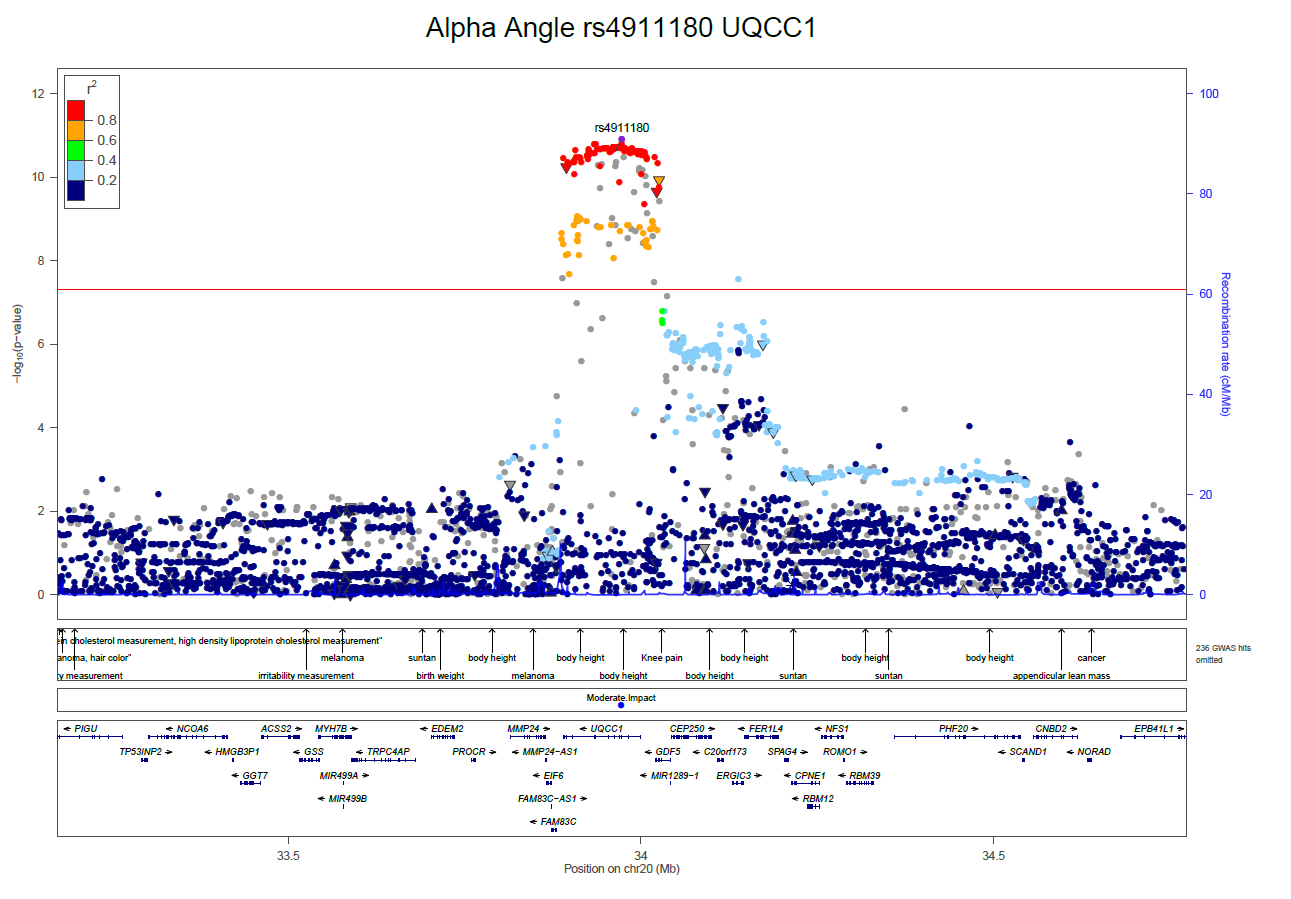


Supplementary Figure 4g. Locus zoom plot rs4911180 (*UQCC1*)


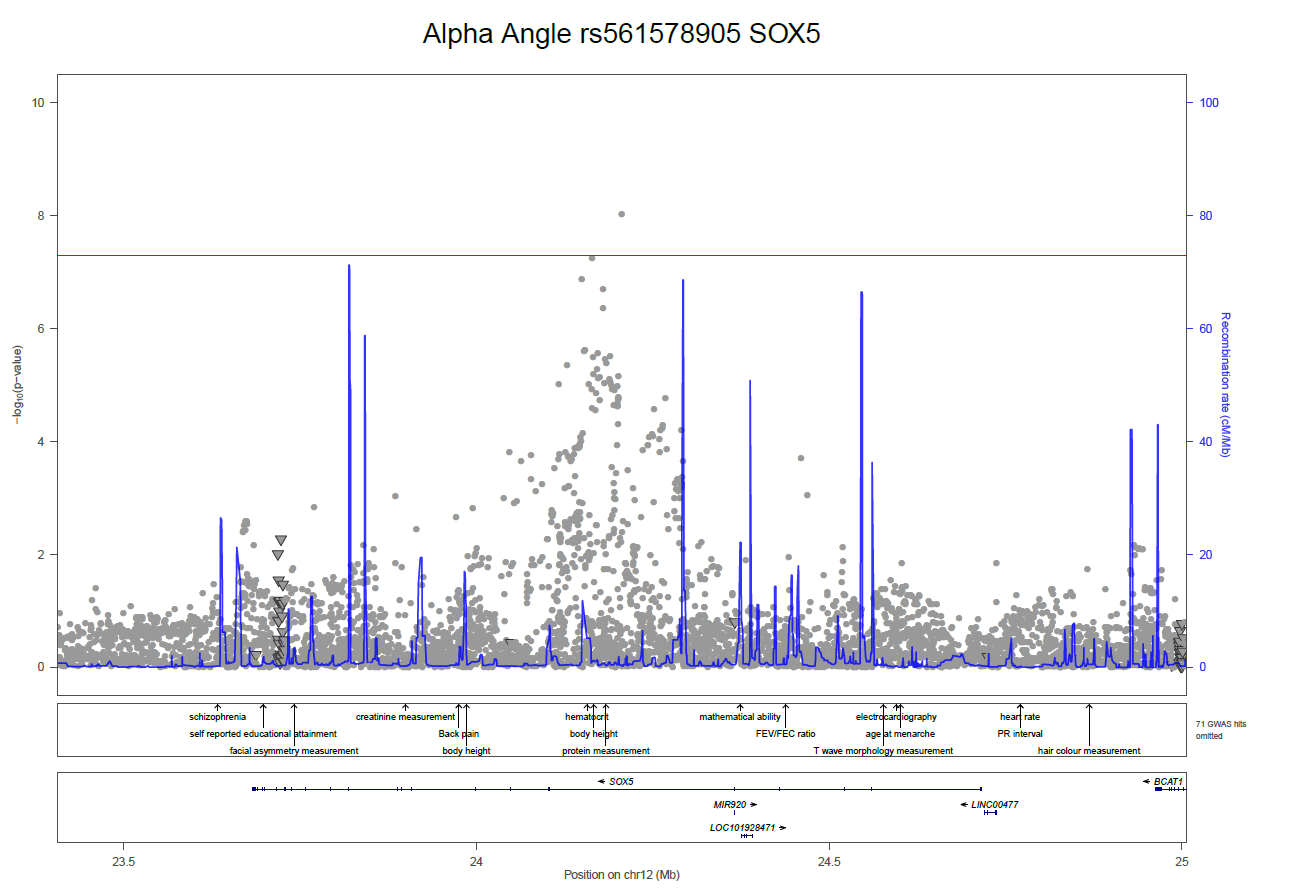


Supplementary Figure 4h. Locus zoom plot rs561578905 (*SOX5*)
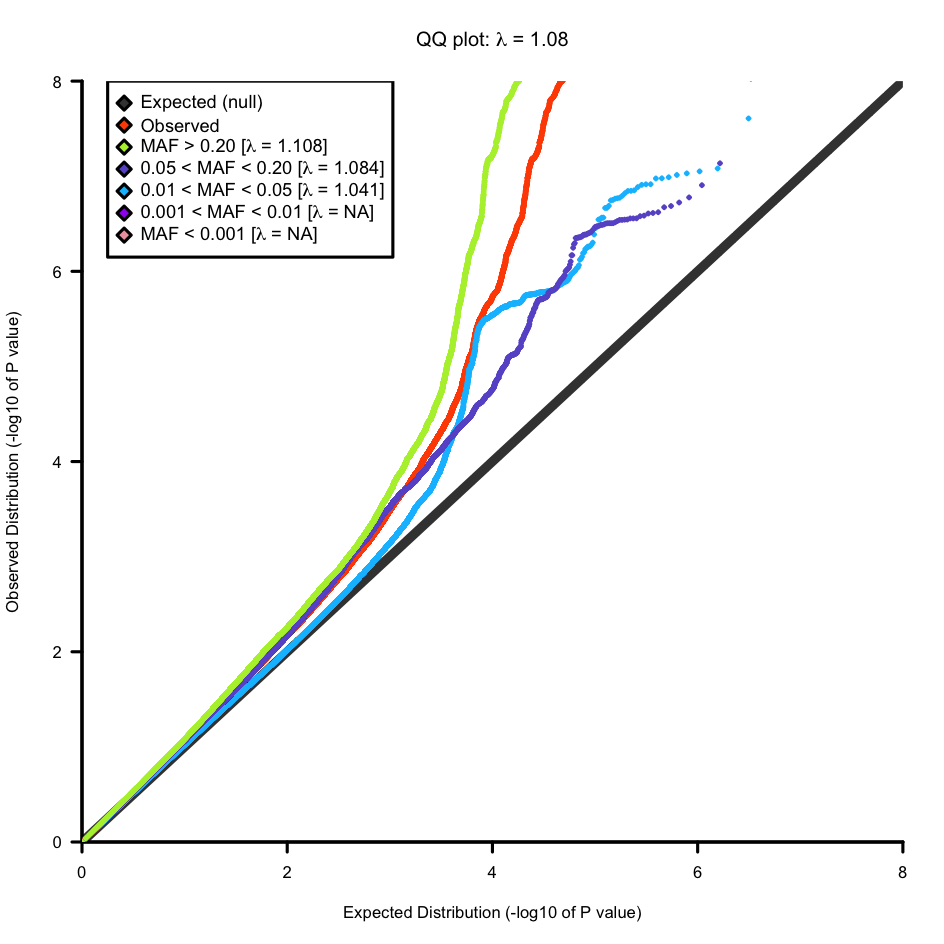


Supplementary Figure 5. QQ plot for alpha angle GWAS meta-analysis


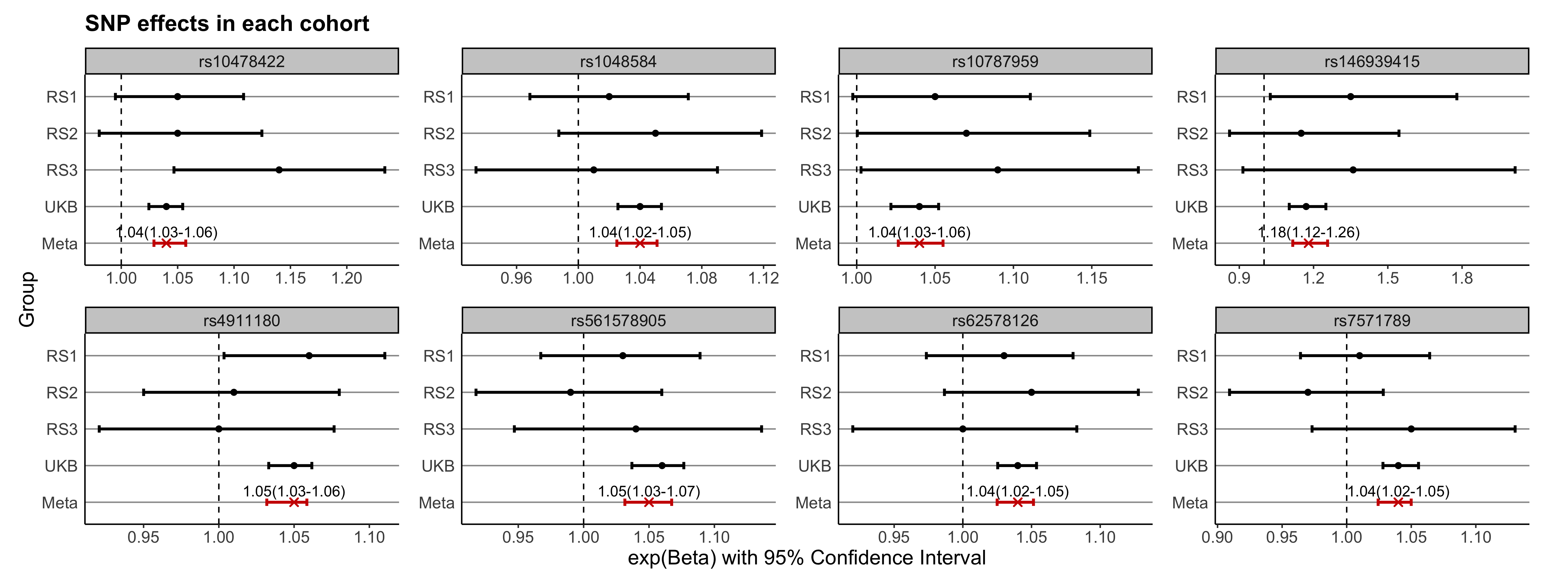


Supplementary Figure 6. A Forest Plot for each independent SNP.

The SNP effects from each cohort are displayed. The exponentiated beta is displayed to aid visualisation. The heterogeneity statistic (I^2^) was zero for all SNPs apart from rs7571789 (I^2^ = 53, P-value = 0.09), rs10478422 (I^2^ = 33, P = 0.21) and rs561578905 (I^2^ = 25, P = 0.26). RS - Rotterdam Study, UKB - UK Biobank, Meta - Meta-analysis.


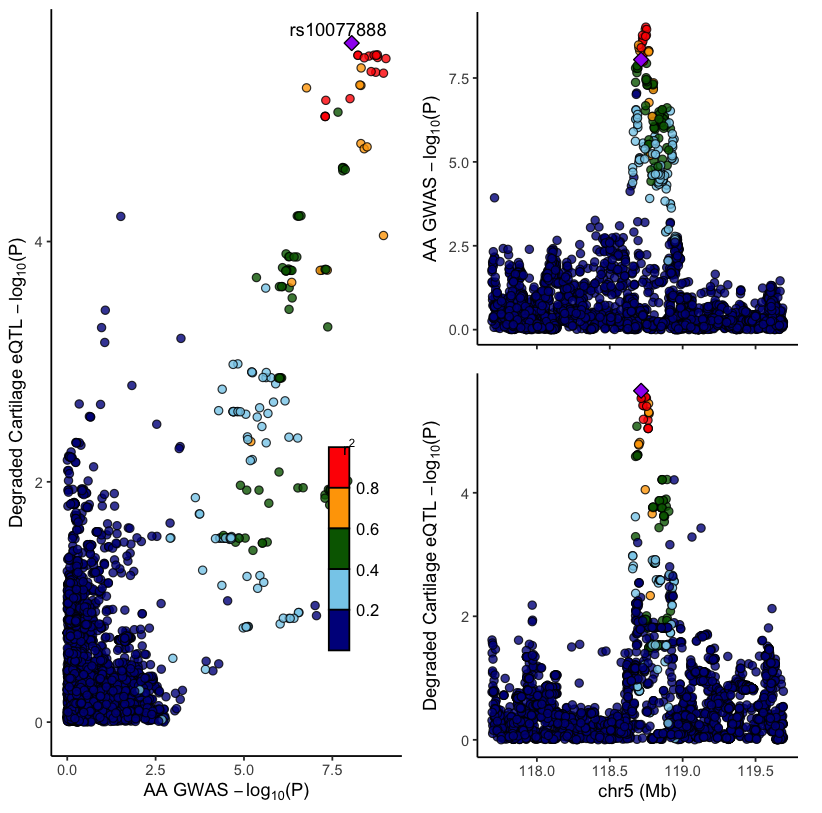


Supplementary Figure 7. A colocalisation plot for *TNFAIP8* expression in highly degraded human cartilage.


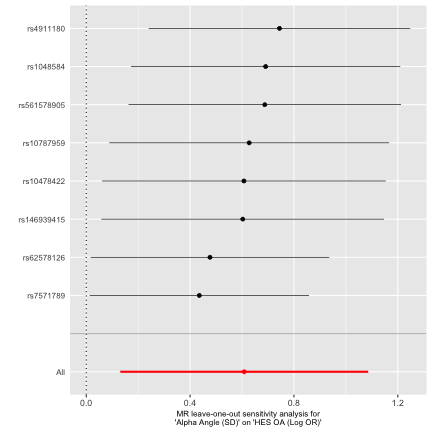


Supplementary Figure 8a. Leave one out analysis comparing alpha angles effect on hip osteoarthritis.


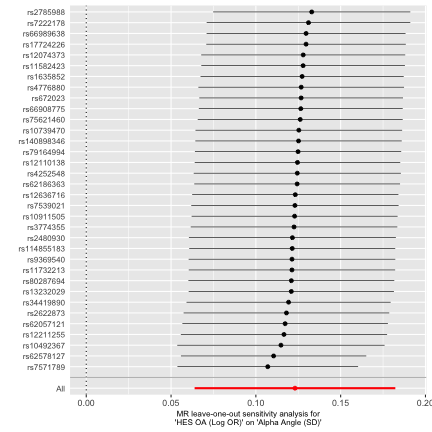


Supplementary Figure 8b. Leave one out analysis comparing hip osteoarthritis effect on alpha angle.


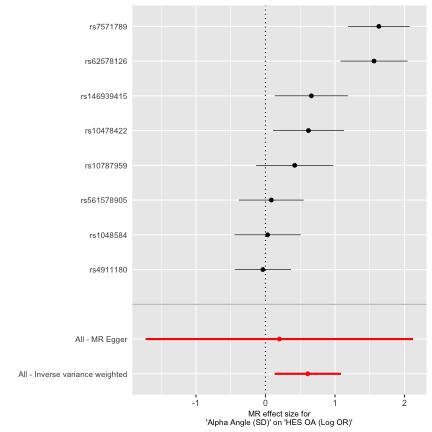


Supplementary Figure 9a. Single SNP analysis of alpha angles effect on hip osteoarthritis.


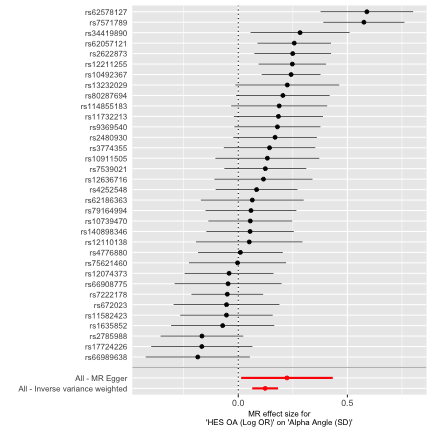


Supplementary Figure 9b. Single SNP analysis of hip osteoarthritis effect on alpha angle.
