# Supplementary material for: A Genome‐Wide Association Study Meta‐Analysis of Alpha Angle Suggests Cam‐Type Morphology May Be a Specific Feature of Hip Osteoarthritis in Older Adults
Source: Arthritis Rheumatol. 2023 Apr 9;75(6):900–9. doi: 10.1002/art.42451 (PMC10374163; doi:10.1002/art.42451)
Supplement: Supplementary file 4 — Supplementary Table 1 Descriptives for the UK Biobank individuals included in this study. Supplementary Table 2. Observational associations between cam morphology and clinical outcomes Supplementary Table 3. Alpha angle GWAS population Supplementary Table 4. COJO results for AA meta‐analysis Supplementary Table 5. Look up of AA SNPs in a UKB Cam GWAS and HOA GWAS Supplementary Table 6. GTEx look up results for the independent alpha angle SNPs Supplementary Table 7. Colocalisation results between each independent AA loci and eQTL data from human cartilage Supplementary Table 8. RegulomeDB results for each independent SNP. Supplementary Table 9. Linkage disequilibrium score regression between AA, UKB HES OA and Meta‐analysis of HOA Supplementary Table 10. Genetic instruments for Hospital diagnosed hip OA obtained from a GWAS of HES OA in UKB, followed by LD clumping after palindromic SNPs were removed. Supplementary Table 11. MR‐CAUSE analysis [file ART-75-900-s001.docx]

|  | Male | Female | Combined |
| --- | --- | --- | --- |
| Demographics | Mean [SD, Range] | Mean [SD, Range] | Mean [SD, Range] |
| Age (years) | 64.3 [7.7, 44-81] | 63.0 [7.4, 45-82] | 63.6 [7.6, 44-82] |
| Height (cm) | 177.2 [6.6, 150-204] | 163.6 [6.4, 135-198] | 170.1 [9.4, 135-204] |
| Weight (kg) | 83.2 [13.4, 47-171] | 68.2 [12.9, 34-169] | 75.4 [15.1, 34-171] |
| *Ethnicity* | Prevalence [%] | Prevalence [%] | Prevalence [%] |
| White | 18,650 [96.7] | 20,394 [96.9] | 39,004 [96.8] |
| Asian | 266 [1.4] | 171 [0.8] | 437 [1.1] |
| Black | 119 [0.6] | 134 [0.6] | 253 [0.6] |
| Mixed heritage | 61 [0.3] | 118 [0.6] | 179[0.4] |
| Chinese | 51 [0.3] | 65 [0.3] | 116 [0.3] |
| Unknown | 147 [0.8] | 161 [0.8] | 308 [0.8] |
| *Hip Shape* | Mean [SD, Range] | Mean [SD, Range] | Mean [SD, Range] |
| Alpha Angle (degrees) | 51.9 [13.1, 35.0-114.1] | 44.0 [5.8, 31.8-115.0] | 47.8 [10.8, 31.8-115.0] |
| *Osteoarthritis* | Prevalence [%] | Prevalence [%] | Prevalence [%] |
| Hip Pain > 3months | 1,193 [6.1] | 2,058 [9.8] | 3,251 [8.1] |
| rHOA grade ≥2 | 2,086 [10.8] | 931 [4.4] | 3,017 [7.5] |
| rHOA grade ≥3 | 509 [2.6] | 191 [0.9] | 700 [1.7] |
| rHOA grade 4 | 113 [0.6] | 44 [0.2] | 157 [0.4] |
| HES OA | 220 [1.1] | 307 [1.5] | 527 [1.3] |
| THR | 106 [0.6] | 153 [0.7] | 259 [0.6] |
| **Total Sample** | 19,294 [47.8%] | 21,043 [52.2%] | 40,337 |

Supplementary Table 1. Descriptives for the UK Biobank individuals included in this study.

SD – standard deviation, rHOA – radiographic hip osteoarthritis, HES OA – hospital diagnosed hip osteoarthritis, THR – total hip replacement

Supplementary Table 2. Observational associations between cam morphology and clinical outcomes

|  | Cam morphology defined as AA ≥60 degrees | | | |
| --- | --- | --- | --- | --- |
|  | Unadjusted | | Adjusted | |
| Cross sectional analyses | OR [95% CI] | *P* | OR [95% CI] | *P* |
| Hip Pain | 1.14 [1.01-1.29] | 0.04 | 1.47 [1.29-1.68] | 6.55E-09 |
| rHOA grade ≥2 | 5.38 [4.92-5.88] | 1.00E-303 | 3.99 [3.63-4.38] | 5.00E-182 |
| rHOA grade ≥3 | 8.70 [7.46-10.15] | 9.00E-167 | 6.71 [5.67-7.95] | 2.00E-107 |
| rHOA grade 4 | 11.08 [8.09-15.18] | 1.10E-50 | 9.14 [6.40-13.04] | 4.07E-34 |
| HES OA | 2.39 [1.91-3.00] | 2.84E-14 | 2.88 [2.25-3.69] | 6.54E-17 |
| Longitudinal analyses | HR [95% CI] | *P* | HR [95% CI] | *P* |
| THR | 2.52 [1.84-3.44] | 5.93E-09 | 3.04 [2.16-4.28] | 1.92E-10 |

Cam (n=3,445 [8.5%]) morphology assessed as a binary outcome. Logistic and Cox proportional hazard model results presented. Adjusted model includes ages, sex, height and weight. OR - odds ratio, CI - confidence interval, rHOA - radiographic hip OA, HR - hazard ratio, P - p-value, HES OA - hospital diagnosed hip osteoarthritis, THR - total hip replacement.

Supplementary Table 3. Alpha angle GWAS population

| Population | Female (n) | Male (n) | Combined (n) |
| --- | --- | --- | --- |
| UKB | 19848 | 18325 | 38173 |
| RS1 | 1688 | 1282 | 2970 |
| RS2 | 971 | 846 | 1817 |
| RS3 | 714 | 540 | 1254 |
| Total | 23221 | 20993 | 44214 |
|  | Female mean age (SD) | Male mean age (SD) | Combined mean age (SD) |
| UKB | 63.0 (7.4) | 64.3 (7.7) | 63.7 (7.6) |
| RS1 | 65.6 (6.8) | 65.3 (6.6) | 65.5 (6.7) |
| RS2 | 64.5 (7.9) | 64.4 (7.5) | 64.4 (7.7) |
| RS3 | 59.6 (3.5) | 59.5 (3.2) | 59.3 (3.4) |
| Combined | 63.1 (7.3) | 64.2 (7.5) | 63.7 (7.4) |

Supplementary Table 4. COJO results for AA meta-analysis

| SNPID | RSID | CHR | BP | C.GENE | EA | NEA | EAF | BETA | SE | P | DIR | N |
| --- | --- | --- | --- | --- | --- | --- | --- | --- | --- | --- | --- | --- |
| 2:70714793:T:C | rs7571789 | 2 | 70714793 | TGFA | T | C | 0.48 | 0.04 | 0.01 | 7.52E-09 | ++-+ | 44214 |
| 5:118752897:A:G | rs455991 | 5 | 118752897 | TNFAIP8 | A | G | 0.70 | -0.04 | 0.01 | 1.13E-09 | ---- | 44214 |
| 6:155578599:A:T | rs1048584 | 6 | 155578599 | TIAM2 | A | T | 0.39 | -0.04 | 0.01 | 7.67E-09 | ---- | 44214 |
| 9:129375338:T:C | rs62578126 | 9 | 129375338 | LMX1B | T | C | 0.37 | -0.04 | 0.01 | 9.00E-09 | ---+ | 44214 |
| 10:121131313:A:G | rs10787959 | 10 | 121131313 | GRK5 | A | G | 0.28 | -0.04 | 0.01 | 1.08E-08 | ---- | 44214 |
| 15:51522210:C:G | rs146939415 | 15 | 51522210 | CYP19A1 | C | G | 0.01 | 0.17 | 0.03 | 2.47E-08 | ++++ | 44214 |
| 20:33972948:A:G | rs4911180 | 20 | 33972948 | UQCC1 | A | G | 0.63 | -0.04 | 0.01 | 1.25E-11 | ---+ | 44214 |

CHR- chromosome, BP - base position, C.Gene - closest gene, EA - effect allele, NEA - non effect allele, EAF - effect allele frequency, SE - standard error, P - p-value, DIR – direction

Supplementary Table 5. Look up of AA SNPs in a UKB Cam GWAS and HOA GWAS

| RSID | EA | NEA | EAF | AA Beta | AA P-value | Cam Beta | Cam P-value | HOA (UKB) logOR | HOA (UKB) P-value |
| --- | --- | --- | --- | --- | --- | --- | --- | --- | --- |
| rs7571789 | T | C | 0.48 | 0.04 | 7.52E-09 | 0.01 | 1.30E-05 | 0.07 | 5.80E-13 |
| rs10478422 | T | C | 0.30 | 0.04 | 9.64E-10 | 0.01 | 1.80E-04 | 0.02 | 0.02 |
| rs1048584 | A | T | 0.39 | -0.04 | 7.67E-09 | -0.01 | 1.10E-04 | -0.001 | 0.91 |
| rs62578126 | T | C | 0.37 | -0.04 | 9.00E-09 | -0.01 | 2.00E-06 | -0.06 | 2.70E-10 |
| rs10787959 | A | G | 0.28 | -0.04 | 1.08E-08 | -0.01 | 1.80E-03 | -0.02 | 0.10 |
| rs561578905 | A | C | 0.27 | 0.05 | 3.37E-08 | 0.01 | 6.40E-05 | 0.005 | 0.71 |
| rs146939415 | C | G | 0.01 | 0.17 | 2.47E-08 | 0.04 | 6.20E-05 | 0.11 | 0.01 |
| rs4911180 | A | G | 0.63 | -0.04 | 1.25E-11 | -0.01 | 4.60E-07 | 0.002 | 0.84 |

Each independent SNP identified in the AA meta-analysis was subsequently looked up in a UKB GWAS of cam morphology. Cam morphology was defined as AA ≥60degrees.

Supplementary Table 6. GTEx look up results for the independent alpha angle SNPs

| Gene Symbol | SNP Id | P-Value | NES | Tissue | PP | Median TPM |
| --- | --- | --- | --- | --- | --- | --- |
| TGFA | rs7571789 | 2.40E-32 | -0.65 | Testis | 0.00 | 2.02 |
| TGFA | rs7571789 | 3.60E-10 | 0.29 | Brain - Cortex |  |  |
| TGFA | rs7571789 | 7.90E-09 | 0.28 | Brain - Hypothalamus |  |  |
| TGFA | rs7571789 | 1.80E-07 | 0.21 | Brain - Caudate (basal ganglia) |  |  |
| TGFA | rs7571789 | 0.0000048 | 0.25 | Brain - Amygdala |  |  |
| TGFA | rs7571789 | 0.000011 | 0.2 | Brain - Frontal Cortex (BA9) |  |  |
| TGFA | rs7571789 | 0.000011 | 0.24 | Brain - Anterior cingulate cortex (BA24) |  |  |
| Gene Symbol | SNP Id | P-Value | NES | Tissue | PP4 | Median TPM |
| TNFAIP8 | rs10478422 | 3.90E-19 | 0.27 | Cells - Cultured fibroblasts | 0.97 | 4.92 |
| HSD17B4 | rs10478422 | 1.10E-14 | 0.21 | Nerve - Tibial |  |  |
| HSD17B4 | rs10478422 | 3.20E-10 | 0.11 | Thyroid |  |  |
| HSD17B4 | rs10478422 | 1.40E-09 | 0.19 | Stomach |  |  |
| HSD17B4 | rs10478422 | 5.40E-08 | 0.12 | Cells - Cultured fibroblasts | 0.93 | 78.88 |
| HSD17B4 | rs10478422 | 1.30E-07 | 0.16 | Esophagus - Muscularis |  |  |
| FAM170A | rs10478422 | 0.0000036 | 0.27 | Thyroid |  |  |
| HSD17B4 | rs10478422 | 0.000011 | 0.12 | Breast - Mammary Tissue |  |  |
| FAM170A | rs10478422 | 0.000019 | 0.41 | Liver |  |  |
| TNFAIP8 | rs10478422 | 0.000021 | -0.19 | Spleen |  |  |
| HSD17B4 | rs10478422 | 0.00003 | 0.18 | Esophagus - Gastroesophageal Junction |  |  |
| HSD17B4 | rs10478422 | 0.000039 | 0.15 | Artery - Tibial |  |  |
| TNFAIP8 | rs10478422 | 0.00019 | -0.072 | Whole Blood |  |  |
| Gene Symbol | SNP Id | P-Value | NES | Tissue | PP4 | Median TPM |
| RP11-477D19.2 | rs1048584 | 1.20E-93 | -0.77 | Thyroid | 0.86 | 4.65 |
| RP11-477D19.2 | rs1048584 | 1.10E-65 | -0.69 | Skin - Sun Exposed (Lower leg) |  |  |
| RP11-477D19.2 | rs1048584 | 7.30E-51 | -0.93 | Brain - Cerebellum |  |  |
| RP11-477D19.2 | rs1048584 | 1.40E-50 | -0.65 | Skin - Not Sun Exposed (Suprapubic) |  |  |
| RP11-477D19.2 | rs1048584 | 1.10E-47 | -0.92 | Brain - Cerebellar Hemisphere |  |  |
| RP11-477D19.2 | rs1048584 | 5.20E-40 | -0.68 | Testis |  |  |
| RP11-477D19.2 | rs1048584 | 2.50E-34 | -0.66 | Adrenal Gland |  |  |
| RP11-477D19.2 | rs1048584 | 5.60E-32 | -0.55 | Colon - Transverse |  |  |
| RP11-477D19.2 | rs1048584 | 9.90E-31 | -0.72 | Pituitary |  |  |
| RP11-477D19.2 | rs1048584 | 9.90E-27 | -0.42 | Artery - Tibial |  |  |
| RP11-477D19.2 | rs1048584 | 2.80E-24 | -0.4 | Breast - Mammary Tissue |  |  |
| RP11-477D19.2 | rs1048584 | 2.50E-23 | -0.48 | Colon - Sigmoid |  |  |
| RP11-477D19.2 | rs1048584 | 3.50E-23 | -0.39 | Esophagus - Mucosa |  |  |
| TFB1M | rs1048584 | 3.70E-23 | -0.27 | Cells - Cultured fibroblasts | 0.66 | 13.18 |
| RP11-477D19.2 | rs1048584 | 7.80E-22 | -0.68 | Brain - Hypothalamus |  |  |
| RP11-477D19.2 | rs1048584 | 8.40E-22 | -0.35 | Whole Blood |  |  |
| RP11-477D19.2 | rs1048584 | 7.70E-20 | -0.41 | Stomach |  |  |
| RP11-477D19.2 | rs1048584 | 2.70E-17 | -0.58 | Brain - Frontal Cortex (BA9) |  |  |
| RP11-477D19.2 | rs1048584 | 3.30E-17 | -0.41 | Heart - Atrial Appendage |  |  |
| RP11-477D19.2 | rs1048584 | 6.00E-17 | -0.6 | Brain - Nucleus accumbens (basal ganglia) |  |  |
| RP11-477D19.2 | rs1048584 | 1.00E-16 | -0.36 | Nerve - Tibial |  |  |
| RP11-477D19.2 | rs1048584 | 1.20E-16 | -0.34 | Cells - Cultured fibroblasts | 0.9 | 1.63 |
| RP11-477D19.2 | rs1048584 | 6.20E-16 | -0.53 | Brain - Cortex |  |  |
| RP11-477D19.2 | rs1048584 | 8.90E-16 | -0.38 | Artery - Aorta |  |  |
| TFB1M | rs1048584 | 1.60E-15 | -0.32 | Thyroid |  |  |
| RP11-477D19.2 | rs1048584 | 1.90E-15 | -0.63 | Small Intestine - Terminal Ileum |  |  |
| RP11-477D19.2 | rs1048584 | 8.40E-15 | -0.55 | Spleen |  |  |
| RP11-477D19.2 | rs1048584 | 2.60E-14 | -0.48 | Prostate |  |  |
| RP11-477D19.2 | rs1048584 | 4.20E-14 | -0.53 | Brain - Caudate (basal ganglia) |  |  |
| CLDN20 | rs1048584 | 1.00E-13 | 0.38 | Adipose - Subcutaneous | 0.58 | 0.44 |
| RP11-477D19.2 | rs1048584 | 2.30E-13 | -0.3 | Adipose - Subcutaneous |  |  |
| RP11-477D19.2 | rs1048584 | 4.30E-13 | -0.27 | Esophagus - Muscularis |  |  |
| CLDN20 | rs1048584 | 6.20E-13 | 0.61 | Cells - EBV-transformed lymphocytes |  |  |
| TFB1M | rs1048584 | 3.30E-12 | -0.25 | Heart - Left Ventricle |  |  |
| RP11-477D19.2 | rs1048584 | 7.70E-12 | -0.28 | Lung |  |  |
| TFB1M | rs1048584 | 3.70E-11 | -0.27 | Colon - Transverse |  |  |
| CLDN20 | rs1048584 | 4.30E-11 | 0.31 | Skin - Sun Exposed (Lower leg) |  |  |
| CLDN20 | rs1048584 | 7.20E-11 | 0.35 | Esophagus - Mucosa |  |  |
| RP11-477D19.2 | rs1048584 | 9.50E-11 | -0.28 | Esophagus - Gastroesophageal Junction |  |  |
| RP11-477D19.2 | rs1048584 | 1.20E-10 | -0.5 | Brain - Anterior cingulate cortex (BA24) |  |  |
| RP11-477D19.2 | rs1048584 | 1.70E-10 | -0.52 | Minor Salivary Gland |  |  |
| RP11-477D19.2 | rs1048584 | 2.20E-10 | -0.29 | Heart - Left Ventricle |  |  |
| RP11-477D19.2 | rs1048584 | 4.50E-10 | -0.58 | Uterus |  |  |
| TFB1M | rs1048584 | 5.40E-10 | -0.42 | Adrenal Gland |  |  |
| RP11-477D19.2 | rs1048584 | 6.10E-10 | -0.37 | Pancreas |  |  |
| RP11-477D19.2 | rs1048584 | 6.60E-10 | -0.61 | Brain - Spinal cord (cervical c-1) |  |  |
| RP11-477D19.2 | rs1048584 | 1.60E-09 | -0.47 | Ovary |  |  |
| RP11-477D19.2 | rs1048584 | 2.80E-09 | -0.48 | Brain - Hippocampus |  |  |
| TIAM2 | rs1048584 | 4.00E-09 | 0.16 | Artery - Tibial | 0.73 | 2.50 |
| TFB1M | rs1048584 | 1.70E-08 | -0.26 | Esophagus - Mucosa |  |  |
| TFB1M | rs1048584 | 3.90E-08 | -0.37 | Pituitary |  |  |
| RP11-477D19.2 | rs1048584 | 4.30E-08 | -0.24 | Adipose - Visceral (Omentum) |  |  |
| RP11-477D19.2 | rs1048584 | 1.30E-07 | -0.52 | Brain - Amygdala |  |  |
| RP11-477D19.2 | rs1048584 | 1.70E-07 | -0.46 | Brain - Putamen (basal ganglia) |  |  |
| CLDN20 | rs1048584 | 5.30E-07 | 0.25 | Cells - Cultured fibroblasts | 0.87 | 0.51 |
| CLDN20 | rs1048584 | 8.50E-07 | 0.29 | Esophagus - Muscularis |  |  |
| RP11-477D19.2 | rs1048584 | 9.80E-07 | -0.3 | Artery - Coronary |  |  |
| RP11-477D19.2 | rs1048584 | 0.0000015 | -0.49 | Brain - Substantia nigra |  |  |
| CLDN20 | rs1048584 | 0.0000018 | 0.2 | Thyroid |  |  |
| CLDN20 | rs1048584 | 0.0000021 | 0.24 | Artery - Tibial |  |  |
| TFB1M | rs1048584 | 0.0000024 | -0.21 | Stomach |  |  |
| CLDN20 | rs1048584 | 0.0000029 | 0.16 | Nerve - Tibial |  |  |
| CLDN20 | rs1048584 | 0.0000042 | 0.37 | Ovary |  |  |
| CLDN20 | rs1048584 | 0.0000068 | 0.25 | Lung |  |  |
| CLDN20 | rs1048584 | 0.000011 | 0.26 | Artery - Aorta |  |  |
| RP11-477D19.2 | rs1048584 | 0.00002 | -0.2 | Liver |  |  |
| TIAM2 | rs1048584 | 0.000027 | 0.24 | Lung |  |  |
| CLDN20 | rs1048584 | 0.000032 | 0.2 | Muscle - Skeletal |  |  |
| TFB1M | rs1048584 | 0.000052 | -0.16 | Heart - Atrial Appendage |  |  |
| CLDN20 | rs1048584 | 0.000065 | 0.18 | Skin - Not Sun Exposed (Suprapubic) |  |  |
| TIAM2 | rs1048584 | 0.00009 | 0.13 | Thyroid |  |  |
| Gene Symbol | SNP Id | P-Value | NES | Tissue | PP4 | Median TPM |
| LMX1B | rs62578126 | 7.80E-15 | -0.32 | Adipose - Subcutaneous | 0.96 | 0.82 |
| LMX1B | rs62578126 | 1.30E-08 | -0.23 | Nerve - Tibial |  |  |
| LMX1B | rs62578126 | 2.00E-07 | -0.21 | Artery - Tibial |  |  |
| RP11-123K19.1 | rs62578126 | 3.80E-07 | -0.28 | Adipose - Subcutaneous | 0.25 | 0.15 |
| LMX1B | rs62578126 | 0.000023 | -0.24 | Adipose - Visceral (Omentum) |  |  |
| LMX1B | rs62578126 | 0.00015 | -0.17 | Thyroid |  |  |
| Gene Symbol | SNP Id | P-Value | NES | Tissue | PP4 | Median TPM |
| GRK5 | rs10787959 | 0.00017 | 0.075 | Skin - Sun Exposed (Lower leg) | 0.48 | 20.20 |
| Gene Symbol | SNP Id | P-Value | NES | Tissue | PP4 | Median TPM |
| N/A | rs561578905 | N/A | N/A | N/A | N/A | N/A |
| Gene Symbol | SNP Id | P-Value | NES | Tissue | PP4 | Median TPM |
| GLDN | rs146939415 | 0.0000023 | 0.46 | Cells - Cultured fibroblasts | 0.00 | 2.4 |
| Gene Symbol | SNP Id | P-Value | NES | Tissue | PP4 | Median TPM |
| UQCC1 | rs4911180 | 4.80E-66 | 0.55 | Cells - Cultured fibroblasts | 0.00 | 20.98 |
| UQCC1 | rs4911180 | 1.30E-38 | 0.39 | Skin - Sun Exposed (Lower leg) |  |  |
| UQCC1 | rs4911180 | 2.80E-36 | 0.24 | Muscle - Skeletal |  |  |
| UQCC1 | rs4911180 | 5.20E-30 | 0.29 | Skin - Not Sun Exposed (Suprapubic) |  |  |
| UQCC1 | rs4911180 | 3.80E-22 | 0.26 | Esophagus - Mucosa |  |  |
| UQCC1 | rs4911180 | 4.00E-22 | 0.33 | Adipose - Subcutaneous |  |  |
| UQCC1 | rs4911180 | 1.90E-20 | 0.29 | Lung |  |  |
| UQCC1 | rs4911180 | 4.30E-19 | 0.22 | Whole Blood |  |  |
| UQCC1 | rs4911180 | 7.40E-18 | 0.23 | Adipose - Visceral (Omentum) |  |  |
| GDF5 | rs4911180 | 1.00E-15 | -0.48 | Pituitary | 0.03 | 0.69 |
| FAM83C | rs4911180 | 4.90E-15 | 0.16 | Skin - Sun Exposed (Lower leg) | 0 | 76.09 |
| GDF5 | rs4911180 | 4.90E-14 | -0.43 | Esophagus - Gastroesophageal Junction |  |  |
| UQCC1 | rs4911180 | 1.10E-13 | 0.26 | Stomach |  |  |
| UQCC1 | rs4911180 | 1.40E-13 | 0.27 | Breast - Mammary Tissue |  |  |
| RPL36P4 | rs4911180 | 4.20E-13 | -0.37 | Artery - Tibial | N/A | 0.77 |
| RPL36P4 | rs4911180 | 6.60E-13 | -0.42 | Cells - Cultured fibroblasts | 0 | 2.07 |
| RPL36P4 | rs4911180 | 4.70E-11 | -0.37 | Adipose - Subcutaneous |  |  |
| UQCC1 | rs4911180 | 5.20E-11 | 0.21 | Nerve - Tibial |  |  |
| GDF5 | rs4911180 | 1.10E-10 | 0.26 | Lung |  |  |
| RPL36P4 | rs4911180 | 1.30E-10 | -0.38 | Nerve - Tibial |  |  |
| RPL36P4 | rs4911180 | 1.30E-10 | -0.44 | Breast - Mammary Tissue |  |  |
| CPNE1 | rs4911180 | 1.70E-10 | 0.3 | Adipose - Subcutaneous | N/A | 105.90 |
| GDF5 | rs4911180 | 2.20E-10 | -0.38 | Colon - Sigmoid |  |  |
| CPNE1 | rs4911180 | 2.50E-10 | 0.21 | Whole Blood |  |  |
| GDF5 | rs4911180 | 2.90E-10 | -0.28 | Thyroid |  |  |
| CEP250 | rs4911180 | 3.00E-10 | -0.21 | Esophagus - Mucosa | 0 | 5.46 |
| RPL36P4 | rs4911180 | 6.40E-10 | -0.36 | Lung |  |  |
| RPL36P4 | rs4911180 | 7.30E-10 | -0.36 | Skin - Not Sun Exposed (Suprapubic) |  |  |
| RPL36P4 | rs4911180 | 9.30E-10 | -0.3 | Muscle - Skeletal |  |  |
| RPL36P4 | rs4911180 | 1.10E-09 | -0.35 | Thyroid |  |  |
| CPNE1 | rs4911180 | 1.10E-09 | 0.37 | Testis |  |  |
| GDF5 | rs4911180 | 2.50E-09 | -0.32 | Esophagus - Muscularis |  |  |
| RPL36P4 | rs4911180 | 2.60E-09 | -0.34 | Skin - Sun Exposed (Lower leg) |  |  |
| UQCC1 | rs4911180 | 4.00E-09 | 0.17 | Thyroid |  |  |
| UQCC1 | rs4911180 | 4.30E-09 | 0.13 | Heart - Atrial Appendage |  |  |
| UQCC1 | rs4911180 | 5.50E-09 | 0.25 | Pancreas |  |  |
| FAM83C | rs4911180 | 5.90E-09 | 0.14 | Skin - Not Sun Exposed (Suprapubic) |  |  |
| UQCC1 | rs4911180 | 9.80E-09 | 0.15 | Artery - Tibial |  |  |
| RPL36P4 | rs4911180 | 1.20E-08 | -0.34 | Esophagus - Mucosa |  |  |
| CPNE1 | rs4911180 | 1.30E-08 | 0.25 | Skin - Sun Exposed (Lower leg) |  |  |
| GDF5 | rs4911180 | 1.50E-08 | -0.41 | Brain - Putamen (basal ganglia) |  |  |
| RPL36P4 | rs4911180 | 1.60E-08 | -0.27 | Whole Blood |  |  |
| UQCC1 | rs4911180 | 3.40E-08 | 0.13 | Heart - Left Ventricle |  |  |
| EPB41L1 | rs4911180 | 5.30E-08 | 0.19 | Esophagus - Mucosa |  |  |
| RPL36P4 | rs4911180 | 6.50E-08 | -0.4 | Testis |  |  |
| RPL36P4 | rs4911180 | 7.10E-08 | -0.47 | Adrenal Gland |  |  |
| UQCC1 | rs4911180 | 1.10E-07 | 0.32 | Minor Salivary Gland |  |  |
| RPL36P4 | rs4911180 | 1.30E-07 | -0.4 | Brain - Nucleus accumbens (basal ganglia) |  |  |
| CPNE1 | rs4911180 | 1.40E-07 | 0.23 | Skin - Not Sun Exposed (Suprapubic) |  |  |
| CPNE1 | rs4911180 | 1.50E-07 | 0.27 | Esophagus - Mucosa |  |  |
| CPNE1 | rs4911180 | 1.50E-07 | 0.21 | Lung |  |  |
| UQCC1 | rs4911180 | 2.30E-07 | 0.17 | Colon - Sigmoid |  |  |
| UQCC1 | rs4911180 | 2.40E-07 | 0.19 | Brain - Hippocampus |  |  |
| RPL36P4 | rs4911180 | 2.70E-07 | -0.32 | Adipose - Visceral (Omentum) |  |  |
| RPL36P4 | rs4911180 | 3.00E-07 | -0.38 | Heart - Atrial Appendage |  |  |
| CPNE1 | rs4911180 | 3.20E-07 | 0.25 | Nerve - Tibial |  |  |
| RPL36P4 | rs4911180 | 6.10E-07 | -0.37 | Colon - Transverse |  |  |
| GDF5 | rs4911180 | 6.20E-07 | -0.18 | Colon - Transverse |  |  |
| UQCC1 | rs4911180 | 6.60E-07 | 0.17 | Brain - Caudate (basal ganglia) |  |  |
| RPL36P4 | rs4911180 | 0.0000012 | -0.29 | Esophagus - Muscularis |  |  |
| CPNE1 | rs4911180 | 0.0000013 | 0.34 | Prostate |  |  |
| ERGIC3 | rs4911180 | 0.0000015 | 0.11 | Cells - Cultured fibroblasts | N/A |  |
| RPL36P4 | rs4911180 | 0.0000016 | -0.36 | Esophagus - Gastroesophageal Junction |  |  |
| CPNE1 | rs4911180 | 0.0000017 | 0.35 | Adrenal Gland |  |  |
| RPL36P4 | rs4911180 | 0.0000019 | -0.46 | Spleen |  |  |
| CPNE1 | rs4911180 | 0.000002 | 0.2 | Artery - Tibial |  |  |
| SCAND1 | rs4911180 | 0.0000037 | -0.14 | Artery - Aorta |  |  |
| FER1L4 | rs4911180 | 0.0000062 | 0.22 | Nerve - Tibial |  |  |
| CPNE1 | rs4911180 | 0.0000065 | 0.2 | Thyroid |  |  |
| RP4-614O4.13 | rs4911180 | 0.0000066 | 0.14 | Skin - Sun Exposed (Lower leg) |  |  |
| UQCC1 | rs4911180 | 0.0000087 | 0.45 | Cells - EBV-transformed lymphocytes |  |  |
| RPL36P4 | rs4911180 | 0.00001 | -0.38 | Pancreas |  |  |
| UQCC1 | rs4911180 | 0.000014 | 0.14 | Brain - Frontal Cortex (BA9) |  |  |
| GDF5 | rs4911180 | 0.000018 | -0.38 | Brain - Cortex |  |  |
| CPNE1 | rs4911180 | 0.000019 | 0.23 | Breast - Mammary Tissue |  |  |
| EDEM2 | rs4911180 | 0.000019 | 0.11 | Adipose - Subcutaneous |  |  |
| MAP1LC3A | rs4911180 | 0.000022 | -0.13 | Brain - Hippocampus |  |  |
| CPNE1 | rs4911180 | 0.000023 | 0.19 | Brain - Cerebellar Hemisphere |  |  |
| UQCC1 | rs4911180 | 0.000025 | 0.099 | Esophagus - Muscularis |  |  |
| GDF5 | rs4911180 | 0.000025 | -0.35 | Brain - Frontal Cortex (BA9) |  |  |
| EIF6 | rs4911180 | 0.000025 | 0.065 | Cells - Cultured fibroblasts | N/A |  |
| NFS1 | rs4911180 | 0.000026 | -0.097 | Muscle - Skeletal |  |  |
| CPNE1 | rs4911180 | 0.000026 | 0.17 | Cells - Cultured fibroblasts | N/A |  |
| RPL36P4 | rs4911180 | 0.000028 | -0.3 | Artery - Aorta |  |  |
| UQCC1 | rs4911180 | 0.000031 | 0.1 | Artery - Aorta |  |  |
| UQCC1 | rs4911180 | 0.000032 | 0.2 | Spleen |  |  |
| ERGIC3 | rs4911180 | 0.000033 | -0.096 | Nerve - Tibial |  |  |
| CPNE1 | rs4911180 | 0.000036 | 0.14 | Muscle - Skeletal |  |  |
| MMP24-AS1 | rs4911180 | 0.000037 | 0.11 | Cells - Cultured fibroblasts | N/A |  |
| RPL36P4 | rs4911180 | 0.000037 | -0.3 | Heart - Left Ventricle |  |  |
| UQCC1 | rs4911180 | 0.000038 | 0.22 | Adrenal Gland |  |  |
| RP4-614O4.13 | rs4911180 | 0.000038 | 0.17 | Breast - Mammary Tissue |  |  |
| CPNE1 | rs4911180 | 0.00005 | 0.19 | Brain - Cerebellum |  |  |
| MMP24-AS1 | rs4911180 | 0.00005 | 0.13 | Muscle - Skeletal |  |  |
| UQCC1 | rs4911180 | 0.00005 | 0.11 | Brain - Nucleus accumbens (basal ganglia) |  |  |
| EDEM2 | rs4911180 | 0.000053 | 0.14 | Cells - Cultured fibroblasts | N/A |  |
| FAM83C | rs4911180 | 0.000055 | 0.21 | Prostate |  |  |
| RPL36P4 | rs4911180 | 0.000064 | -0.36 | Prostate |  |  |
| FAM83C | rs4911180 | 0.000068 | 0.11 | Esophagus - Mucosa |  |  |
| EPB41L1 | rs4911180 | 0.000091 | -0.12 | Artery - Aorta |  |  |
| MMP24-AS1 | rs4911180 | 0.000099 | 0.25 | Brain - Putamen (basal ganglia) |  |  |
| RPL36P4 | rs4911180 | 0.00011 | -0.34 | Pituitary |  |  |
| CPNE1 | rs4911180 | 0.00015 | 0.23 | Brain - Caudate (basal ganglia) |  |  |
| FER1L4 | rs4911180 | 0.00015 | 0.12 | Skin - Sun Exposed (Lower leg) |  |  |
| TRPC4AP | rs4911180 | 0.00017 | -0.21 | Brain - Cerebellar Hemisphere |  |  |
| MAP1LC3A | rs4911180 | 0.00017 | -0.078 | Artery - Tibial |  |  |
| CEP250 | rs4911180 | 0.00018 | -0.13 | Testis |  |  |
| RP4-614O4.13 | rs4911180 | 0.00019 | 0.18 | Heart - Atrial Appendage |  |  |
| EIF6 | rs4911180 | 0.00021 | -0.064 | Skin - Not Sun Exposed (Suprapubic) |  |  |
| MYH7B | rs4911180 | 0.00022 | -0.08 | Nerve - Tibial |  |  |
| TRPC4AP | rs4911180 | 0.00022 | 0.13 | Esophagus - Gastroesophageal Junction |  |  |
| RP4-614O4.13 | rs4911180 | 0.00026 | 0.088 | Whole Blood |  |  |
| CEP250 | rs4911180 | 0.00027 | -0.086 | Artery - Tibial |  |  |
| MMP24-AS1 | rs4911180 | 0.00028 | 0.083 | Whole Blood |  |  |
| GDF5OS | rs4911180 | 0.00029 | -0.23 | Testis |  |  |
| EIF6 | rs4911180 | 0.00031 | -0.088 | Muscle - Skeletal |  |  |
| MMP24-AS1 | rs4911180 | 0.00038 | 0.17 | Testis |  |  |
| RP4-614O4.13 | rs4911180 | 0.00066 | 0.088 | Cells - Cultured fibroblasts | N/A |  |

Colocalisation was done with GTEx v7 as that corresponds with hg19 mapping which was used for the GWAS using LocusFocus (https://locusfocus.research.sickkids.ca). All genes within 200kb of the sentinel SNP were examined for eQTL colocalisation using the COLOC2 package. N/A - insufficient data to perform colocalisation, NES - normalised effect sizes, TPM - transcripts per million

Supplementary Table 7. Colocalisation results between each independent AA loci and eQTL data from human cartilage

| Highgrade (unhealthy) tissue | |  |  |
| --- | --- | --- | --- |
| GWASInput | eQTLGene | nsnps | PP |
| rs7571789 | CYP26B1 | 3055 | 0.00 |
| rs7571789 | SNRNP27 | 3720 | 0.00 |
| rs1047842 | TNFAIP8 | 3817 | 0.97 |
| rs6257812 | RPL12 | 2847 | 0.00 |
| rs6257812 | ST6GALN | 2263 | 0.00 |
| rs6257812 | URM1 | 2403 | 0.00 |
| rs6257812 | ZNF79 | 2838 | 0.00 |
| rs1078795 | NANOS1 | 3778 | 0.00 |
| rs1078795 | PDZD8 | 3164 | 0.00 |
| rs1469394 | CYP19A1 | 3506 | 0.02 |
| rs1469394 | GLDN | 3395 | 0.11 |
| rs4911180 | CPNE1 | 2111 | 0.00 |
| rs4911180 | EDEM2 | 2344 | 0.00 |
| rs4911180 | PROCR | 2336 | 0.00 |
| rs4911180 | RALY | 2272 | 0.00 |
| rs4911180 | SCAND1 | 1974 | 0.00 |
| rs4911180 | ZNF341 | 2416 | 0.00 |
| Lowgrade (healthy) tissue | |  |  |
| GWASInput | eQTLGene | nsnps | PP |
| rs1047842 | DTWD2 | 3802 | 0.00 |
| rs1048584 | TFB1M | 3996 | 0.61 |
| rs6257812 | RPL12 | 2775 | 0.00 |
| rs6257812 | ZNF79 | 2767 | 0.00 |
| rs1078795 | SFXN4 | 3950 | 0.00 |
| rs4911180 | CPNE1 | 2061 | 0.00 |
| rs4911180 | UQCC1 | 2138 | 0.01 |
| rs4911180 | ZNF341 | 2350 | 0.00 |
| rs1078795 | PDZD8 | 3164 | 0.00 |
| rs1469394 | CYP19A1 | 3506 | 0.02 |
| rs1469394 | GLDN | 3395 | 0.11 |
| rs4911180 | CPNE1 | 2111 | 0.00 |
| rs4911180 | EDEM2 | 2344 | 0.00 |
| rs4911180 | PROCR | 2336 | 0.00 |
| rs4911180 | RALY | 2272 | 0.00 |
| rs4911180 | SCAND1 | 1974 | 0.00 |
| rs4911180 | ZNF341 | 2416 | 0.00 |

eQTL colocalisation was done on high grade (unhealthy) and low grade (healthy) tissue. 1mb searched either side of the sentinel SNP. If there was no eQTL data from 1mb either side of the sentinel SNP then no results are listed. PP – posterior probability

Supplementary Table 8. RegulomeDB results for each independent SNP.

| **Chr** | **rsids** | **probability** | **ranking** | **ChIP** | **DNase** | **Footprint** | **Footprint matched** | **IC matched max** | **IC max** | **PWM** | **PWM matched** | **QTL** |
| --- | --- | --- | --- | --- | --- | --- | --- | --- | --- | --- | --- | --- |
| **2** | rs7571789 | 0.59 | 5 | TRUE | FALSE | FALSE | FALSE | 0.00 | 0.00 | FALSE | FALSE | FALSE |
| **5** | rs10478422 | 0.61 | 4 | TRUE | TRUE | FALSE | FALSE | 0.00 | 0.00 | FALSE | FALSE | FALSE |
| **6** | rs1048584 | 0.18 | 7 | FALSE | FALSE | FALSE | FALSE | 0.00 | 0.00 | FALSE | FALSE | FALSE |
| **9** | rs62578126 | 0.70 | 4 | TRUE | TRUE | TRUE | FALSE | 0.00 | 0.00 | FALSE | FALSE | FALSE |
| **10** | rs10787959 | 0.99 | 3a | TRUE | TRUE | FALSE | FALSE | 0.00 | 1.04 | TRUE | FALSE | FALSE |
| **12** | rs561578905 | 0.51 | 6 | FALSE | FALSE | TRUE | FALSE | 0.00 | 1.84 | TRUE | FALSE | FALSE |
| **15** | rs146939415 | 1.00 | 2a | TRUE | TRUE | TRUE | TRUE | 0.67 | 0.67 | TRUE | TRUE | FALSE |
| **20** | rs4911180 | 0.13 | 5 | FALSE | TRUE | FALSE | FALSE | 0.00 | 0.00 | FALSE | FALSE | FALSE |

RegulomeDB annotates SNPS with known and predicted regulatory elements in non-coding regions of the genome. A probability and ranking score for each SNP is given. The higher the probability the more likely the SNP is a non-coding regulatory SNP.

ChIP - ChIP-seq signal, DNase - DNase-seq signal, IC - information content change, PWM - position-weight matrix for transcription factor binding, QTL - quantitative trait loci

Supplementary Table 9. Linkage disequilibrium score regression between AA, UKB HES OA and Meta-analysis of HOA

| Trait 1 | Trait 2 | rg [95% CI] | p-value |
| --- | --- | --- | --- |
| UKB AA | RS AA | 0.57 [0.05-1.09] | 0.03 |
| AA Meta-analysis | HOA (UKB) | 0.26 [0.10-0.43] | 1.60E-03 |
| AA Meta-analysis | THR | 0.20 [0.09-0.32] | 6.00E-04 |
| AA Meta-analysis | mJSW (meta-analysis) | -0.31 [-0.46--0.15] | 1.00E-04 |
| AA Meta-analysis | mJSW (X-ray only) | -0.31 [-0.58--0.03] | 0.03 |
| AA Meta-analysis | Acute hip pain | -0.04 [-0.20-0.11] | 0.61 |
| AA Meta-analysis | Chronic hip pain | -0.07 [-0.23-0.09] | 0.37 |
| AA Meta-analysis | Fracture | -0.06 [-0.20-0.07] | 0.36 |
| AA Meta-analysis | Height | 0.09 [-0.01-0.19] | 0.08 |
| AA Meta-analysis | BMI | 0.11 [0.03-0.19] | 0.01 |
| AA Meta-analysis | eBMD | -0.11 [-0.20--0.02] | 0.01 |
| AA Meta-analysis | FN BMD | -0.11 [-0.26-0.05] | 0.19 |
| AA Meta-analysis | Lumbar BMD | -0.04 [-0.20-0.11] | 0.61 |

Rg – Genetic correlation coefficient, UKB - UK Biobank, AA - Alpha Angle, RS - Rotterdam Study, HOA - Hip Osteoarthritis, THR - total hip replacement, mJSW - minimum joint space width, BMI - body mass index, eBMD - estimated bone mineral density, FN BMD - femoral neck bone mineral density

Supplementary Table 10. Genetic instruments for Hospital diagnosed hip OA obtained from a GWAS of HES OA in UKB, followed by LD clumping after palindromic SNPs were removed.

| RSID | CHR | BP | EA | NEA | EAF | LogOR | *P* |
| --- | --- | --- | --- | --- | --- | --- | --- |
| rs7539021 | 1 | 183935831 | A | G | 0.39 | -0.07 | 8.60 x 10^-13^ |
| rs11582423 | 1 | 150493925 | C | G | 0.6 | 0.06 | 4.60 x 10^-10^ |
| rs10911505 | 1 | 184049978 | T | C | 0.64 | 0.06 | 2.10 x 10-^08^ |
| rs12074373 | 1 | 118744920 | A | G | 0.66 | -0.07 | 4.10 x 10^-11^ |
| rs2785988 | 1 | 219744138 | C | A | 0.7 | -0.08 | 1.10 x 10^-12^ |
| rs2622873 | 1 | 103466053 | T | C | 0.87 | 0.11 | 1.30 x 10^-14^ |
| rs7571789 | 2 | 70714793 | T | C | 0.48 | 0.07 | 5.80 x 10^-13^ |
| rs62186363 | 2 | 242176429 | G | A | 0.64 | -0.06 | 1.10 x 10^-08^ |
| rs66989638 | 2 | 106689736 | G | A | 0.88 | -0.09 | 1.70 x 10^-08^ |
| rs12636716 | 3 | 189728328 | A | G | 0.48 | 0.06 | 2.40 x 10^-09^ |
| rs3774355 | 3 | 52817778 | G | A | 0.64 | -0.07 | 1.10 x 10^-10^ |
| rs140898346 | 4 | 13063846 | C | T | 0.4 | -0.07 | 2.00 x 10^-11^ |
| rs11732213 | 4 | 1704244 | T | C | 0.8 | 0.08 | 2.40 x 10^-11^ |
| rs12110138 | 5 | 67842092 | A | G | 0.55 | 0.05 | 2.40 x 10^-08^ |
| rs9369540 | 6 | 45148930 | A | G | 0.62 | 0.07 | 1.00 x 10^-11^ |
| rs66908775 | 6 | 7795032 | G | A | 0.84 | -0.07 | 4.20 x 10^-08^ |
| rs80287694 | 6 | 55636940 | A | G | 0.89 | -0.1 | 4.60 x 10^-10^ |
| rs12211255 | 6 | 76188330 | C | A | 0.9 | -0.14 | 1.10 x 10^-18^ |
| rs13232029 | 7 | 50841045 | G | A | 0.22 | 0.07 | 1.50 x 10^-08^ |
| rs1635852 | 7 | 28189411 | T | C | 0.49 | 0.06 | 9.90 x 10^-09^ |
| rs17724226 | 8 | 10968926 | G | A | 0.54 | 0.06 | 5.90 x 10^-09^ |
| rs79164994 | 8 | 130719443 | A | G | 0.77 | 0.08 | 6.00 x 10^-11^ |
| rs672023 | 9 | 36232341 | T | C | 0.38 | 0.06 | 2.30 x 10^-08^ |
| rs10739470 | 9 | 119377717 | T | C | 0.4 | 0.07 | 2.80 x 10^-12^ |
| rs2480930 | 9 | 117842307 | A | G | 0.47 | 0.07 | 4.80 x 10^-12^ |
| rs62578127 | 9 | 129386860 | C | T | 0.63 | 0.06 | 3.10 x 10^-10^ |
| rs34419890 | 11 | 66501624 | T | C | 0.93 | 0.11 | 3.30 x 10^-09^ |
| rs114855183 | 12 | 122606596 | A | G | 0.53 | -0.06 | 5.10 x 10^-10^ |
| rs10492367 | 12 | 28014970 | G | T | 0.81 | -0.13 | 1.70 x 10^-23^ |
| rs62057121 | 17 | 43900760 | G | A | 0.78 | -0.1 | 1.00 x 10^-15^ |
| rs7222178 | 17 | 59652282 | T | A | 0.8 | -0.1 | 3.30 x 10^-16^ |
| rs75621460 | 19 | 41833784 | G | A | 0.97 | -0.2 | 9.80 x 10^-10^ |
| rs4252548 | 19 | 55879672 | C | T | 0.98 | -0.25 | 2.40 x 10^-13^ |
| rs4776880 | 15 | 67364991 | G | A | 0.66 | 0.07 | 5.10 x 10^-12^ |

Supplementary Table 11. MR-CAUSE analysis

|  |  |  |  |  |  |  |  |  |  |  |  |
| --- | --- | --- | --- | --- | --- | --- | --- | --- | --- | --- | --- |
| **Exposure** | **Outcome** | **Model 1** | **Model 2** | **ELPD** | **se** | ***γ*** | **η** | **q** | **q CIL** | **q CIU** | ***P*-value** |
| AA | HOA (UKB) | Null | Sharing | -0.09 | 0.35 | NA | 0.02 | 0.06 | 0.00 | 0.35 | 0.40 |
|  |  |  |  |  |  |  |  |  |  |  |  |
| AA | HOA (UKB) | Null | Causal | -3.08 | 2.09 | 0.01 | 0 | 0.03 | 0.00 | 0.24 | 0.07 |
|  |  |  |  |  |  |  |  |  |  |  |  |
| AA | HOA (UKB) | Sharing | Causal | -2.99 | 1.75 | NA | NA | NA | NA | NA | 0.04 |
|  |  |  |  |  |  |  |  |  |  |  |  |
| HOA (UKB) | AA | Null | Sharing | -3.47 | 1.89 | NA | 1.75 | 0.2 | 0.02 | 0.47 | 0.03 |
|  |  |  |  |  |  |  |  |  |  |  |  |
| HOA (UKB) | AA | Null | Causal | -7.12 | 3.68 | 0.7 | 0.01 | 0.04 | 0.00 | 0.25 | 0.03 |
|  |  |  |  |  |  |  |  |  |  |  |  |
| HOA (UKB) | AA | Sharing | Causal | -3.65 | 1.83 | NA | NA | NA | NA | NA | 0.02 |
|  |  |  |  |  |  |  |  |  |  |  |  |

ELPD - expected log pointwise posterior density; se - standard error; γ (gamma) - estimate of causal effect if causal model is correct; η (eta) - estimate of correlated pleiotropy; q - proportion of effect due to correlated pleiotropy; CIL/U - confidence intervals lower/ upper; NA - non-applicable.
